# Major type IV pilin of *Legionella pneumophila* enhances bacterial iron acquisition/assimilation, independently of its role in piliation

**DOI:** 10.1128/mbio.00596-26

**Published:** 2026-04-24

**Authors:** Armando Barajas, Joshua Mayoral, Nicholas P. Cianciotto

**Affiliations:** 1 Department of Microbiology and Immunology, Northwestern University Medical School 576055, Chicago, Illinois, USA; University of California Berkeley 1438, Berkeley, California, USA

**Keywords:** *L. pneumophila*, type IV pili, major pilin, iron acquisition, biofilm formation, autoaggregation, transformation competence

## Abstract

We identified a locus within the genome of *Legionella pneumophila* strain 130b that encodes two related type IV pilins, PilA1 and PilA2. Based on *in silico* analyses, both proteins were predicted to be major pilins in all *L. pneumophila* strains and nearly all *Legionella* species. Utilizing immunoblot analysis of sheared-cell supernatants, immunofluorescence microscopy, and whole-cell ELISA, we confirmed that PilA2 (but not PilA1) behaves as a major pilin, and mutant analysis documented that PilA2-containing type IV pili (T4P) are required for DNA uptake, and subject to retraction by PilT. Unexpectedly, a *pilA2* mutant, but not its complement, had impaired growth on solid and liquid media depleted of iron, indicating that PilA2 can promote growth in low-iron conditions. However, mutants lacking PilT, PilD, or PilS/PilR did not exhibit this phenotype, implying that PilA2’s newfound role does not involve inclusion into T4P, cleavage/maturation from prepilin to pilin, or the signal transduction pathway that often modulates the expression of pilin, along with other genes. Mutants lacking the FeoB Fe^2+^ transporter, LbtC siderophore transporter, or Ccm cytochrome maturation showed even greater defects on low-iron agar media when *pilA2* mutations were introduced into them, suggesting that PilA2 functions within a novel iron-uptake pathway. Compatible with this, the *pilA2* mutant, but not its complement, exhibited increased resistance to streptonigrin and heightened expression of iron-regulated genes. Finally, although unimpaired for growth within amoebae, the *pilA2* mutant displayed increased biofilm formation. Overall, this study reveals a novel role for a type IV pilin in bacterial growth in iron acquisition/assimilation.

**IMPORTANCE:**

*Legionella pneumophila* is the main cause of Legionnaires’ disease, an increasingly prevalent pneumonia. Prior studies indicated that *L. pneumophila* expresses type IV pili, a common form of pili that is mainly known for mediating DNA uptake, twitching motility, and attachment to biotic and abiotic surfaces. By utilizing a variety of *in silico* and experimental approaches, wehave helped redefine the major structural component (pilin) of the *L. pneumophila* pili. More significantly, we uncovered a heretofore unknown and seemingly novel role for the pilin in *L. pneumophila* growth in low-iron conditions and iron acquisition/assimilation. These findings have broad implications for understanding other bacteria and infectious diseases, as well as offering fresh insight into the roles of pilins in bacterial physiology and the mechanisms of iron assimilation.

## INTRODUCTION

Gram-negative *Legionella pneumophila* is the agent of Legionnaires’ disease (1, 2). In water habitats, *L. pneumophila* persists free-floating, in biofilms, and as a parasite of amoebae (3, 4). After entering the lungs, *L. pneumophila* grows in macrophages, and this is followed by release from spent host cells, extracellular spread, more intracellular infections, inflammation, and tissue destruction (5–8). Factors that promote the virulence or environmental spread of *L. pneumophila* include a type IV secretion system, type II secretion system (T2SS), type I secretion system, polar flagella, quorum-sensing, and iron uptake (9–17).

Many gram-negative and gram-positive bacteria express type IV pili (T4P) (18–24). T4P are mainly made of many copies of a major pilin (PilA) (18, 25). Less abundant are minor pilins (PilY1, PilV, PilW, PilX, and FimT), some of which are at the tip and others along the length of the pili (22, 26–29). Evolutionarily related to T2SS, the T4P apparatus also has an inner membrane platform (PilC) and ATPases (PilB and PilT), alignment proteins (PilM, PilN, PilO, and PilP), a peptidase that cleaves prepilins into mature pilins (PilD), and the outer membrane complex that places the pili at the surface (PilQ, PilF, FimV, and TsaP) (18, 23, 24, 30). T4P extend and retract using the ATPase motors (31–35). Based on the major pilin sequence and other differences in the apparatus, T4P are divided into types a, b, and c (18–20, 36). Collectively, T4P promote many activities, such as DNA uptake (competence), motility across surfaces (twitching), attachment to biotic or abiotic surfaces, and biofilm formation (18–21, 37–66). Although some T4P-expressing bacteria express multiple T4P types, most have one set of apparatus genes to make one pilus type (18, 21, 23, 25, 36, 41, 67). However, some of these species of *Streptococcus*, *Synechocystis,* and *Thermus* encode two major pilins (64, 68–72). The significance of having two major pilins (with one set of apparatus genes) is not well known, although in *Thermus thermophilus*, one major pilin forms “wide” T4P for competence, and the other forms “thin” T4P for twitching (71).

In 1980, electron microscopy (EM) revealed appendages emanating from the surface of 14 strains of *L. pneumophila*, for example, clinical isolates Philadelphia-1 and Togus-1, giving the first clue that *L. pneumophila* has pili (73, 74). In 1998, EM showed that clinical isolate 130b has “long” and “short” pili (75). Genomic data then indicated that *L. pneumophila* has genes that typically encode T4aP (*pilBCD*, *pilMNOPQ*, *pilT, pilF, fimV,* and *pilZ*), implying that at least some of the bacterium’s pili are T4P (76–81). In that 1998 study, *pilE_L_* was said to encode a protein with similarity to the major pilins of T4P-expressing bacteria (75). Mutant analyses indicated that *pilE_L_* (aka *pilE*) is needed for the production of long pili, DNA uptake, twitching, optimal biofilm, and maximal attachment to amoebae, macrophages, and epithelia (75, 77, 81–84). Thus, *pilE* was long thought to encode the major pilin of *L. pneumophila* T4P, with minor pilins encoded nearby (*pilVWX*, *pilY1*, and *fimU*) and elsewhere (*fimT*/*pspA, and lpp1976-1977-1978*) (77, 79, 81, 85, 86). However, in 2021, mutagenesis of strains HL-0709-3014 and Paris aimed at finding more genes involved in DNA uptake found *pilA2*, a previously undefined gene that also had similarity to major pilins (81). Immunofluorescence (IF) microscopy showed PilA2 on the entire length of pili, affirming it to be a major pilin. Further IF and assessments of protein levels led to a reclassification of PilE as a minor pilin linked to PilA2-T4P (81). Thus, PilA2 majorly comprises the long *L. pneumophila* pili used for DNA uptake. The 2021 study also found, next to *pilA2*, another putative major pilin gene, *pilA1*; however, *pilA1* was not needed for DNA uptake (81).

After discovering that the Cas2 nuclease encoded by the CRISPR-Cas locus of strain 130b fosters *L. pneumophila*’s ability to infect amoebae (87, 88), we used proteomic analysis to identify proteins with increased expression in wild type (WT) vs. a *cas2* mutant and uncovered, among other things, a heat shock protein (HspC2) that promotes infection (89). As a follow-up to that analysis, we assessed the *cas2* mutant using RNA-seq and were surprised to find *pilA2*, which had not yet appeared in the literature. Here, we unexpectedly found that PilA2, independently of the T4P apparatus, promotes *L. pneumophila* growth in low iron and iron acquisition/assimilation.

## RESULTS

### Conservation of *pilA2* and *pilA1* among *L. pneumophila* strains and other *Legionella* species

**Fig 1:**
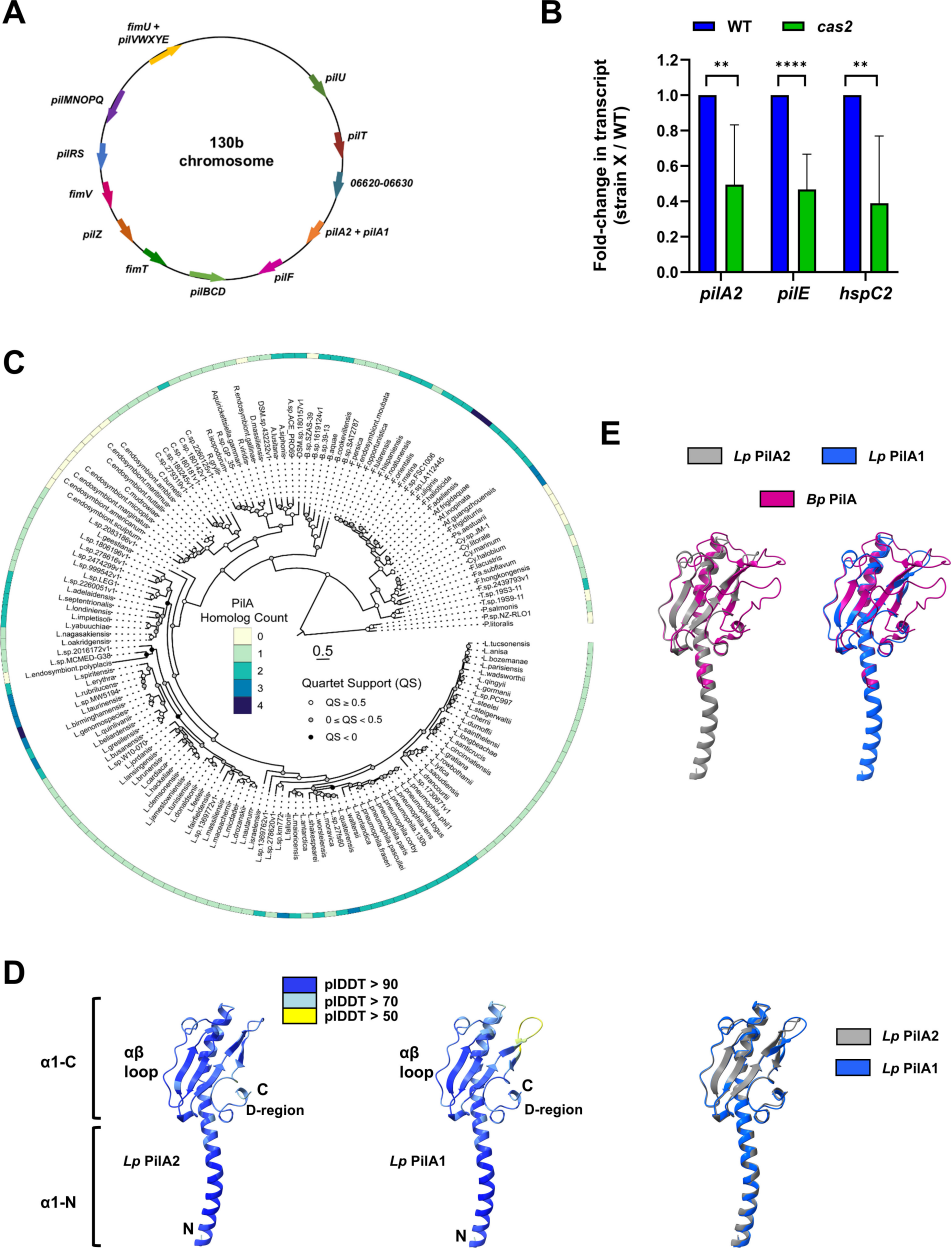
Chromosomal loci, phylogenetic distribution, and predicted structures of PilA2 and PilA1**.** (**A**) Depiction of the *L. pneumophila* strain 130b circular chromosome, highlighting the relative locations of the locus encoding *pilA2* and *pilA1* and the other loci known or predicted to be involved in T4P production or regulation. The locus containing ORFs 06,620, 06,625, and 06,630 corresponds to the *lpp1976–lpp1977–lpp1978* locus of strain Paris. The functions of the proteins encoded by the loci presented here appear in the main text. (**B**) Effect of Cas2 on the expression of *pilA2* and *pilE*. WT 130b and *cas2* mutant NU453 were grown at 37°C in BYE broth to early stationary phase, and RNA was isolated. mRNA levels for *pilA2, pilE*, and, as a control, *hspC2* were determined by qRT-PCR using RNA from six biological replicates (i.e., six separate experiments), each examined in triplicate per strain. Means and standard deviations are presented for the fold changes in the mutant’s transcripts, beside the WT values set to 1.0. Asterisks indicate significant differences between the strains: **, *P* < 0.01; ****, *P* < 0.0001. (**C**) Distribution of genes encoding PilA2/PilA1 homologs within species of *Legionella* and related genera. (Center) A maximum-likelihood phylogenetic tree for unique species in the orders *Berkiellales*, *Coxiellales*, *Diplorickettsiales*, *DSM-16500*, *Francisellales, Legionellales*, and *Piscirickettsiales*. Scale, 1 amino acid substitutions per site. (Outer ring) The presence of one to four genes related to *pilA2/pilA1* are marked by blue-shaded squares, as indicated by the key, whereas the absence of a gene is denoted by white squares, as determined using BLASTP. (**D**) Predicted 3D structures of PilA2 (*Lp* PilA2, left) and PilA1 (*Lp* PilA1, center) (without their signal sequences), as determined by AlphaFold 3. The predicted structures are color-coded in accordance with the levels of confidence (i.e., predicted local distance difference test [pLDDT] values) determined by the program, as indicated by the inserted key. The proteins’ N-termini and C-termini are denoted, as are their N-terminal α-helices (divided into α1-N and α1-C), αβ-loops, and D-regions. An alignment of *Lp* PilA2 (gray) and *Lp* PilA1 (blue), as analyzed using the DALI server, is presented in the right-most panel. (**E**) Alignments of the predicted structures of *Lp* PilA2 (gray, left) and *Lp* PilA1 (blue, right) with the known structure of the major pilin PilA of *B. pseudomallei* (without its 62 N-terminal residues) (*Bp* PilA, magenta) (Protein Data Bank [PDB] ID: 9JW7), giving for PilA2, a root mean square deviation (RMSD) = 1.4, Z = 13.2, and % identity = 45, and for PilA1, an RMSD = 1.7, Z = 12.6, and % identity = 40.

To identify *L. pneumophila* genes besides *hspC2* that are more highly expressed in the presence of Cas2, we had previously grown WT strain 130b and a *cas2* mutant in buffered yeast extract (BYE) broth to early-stationary phase and performed RNA-seq (90). Among the mRNAs that had reduced levels in the mutant were those from previously described *pilE* (75), as well as another type IV pilin-like gene that had not been defined in the literature. Soon after this analysis, *pilA2* from strains Paris and HL-0709-3014 was reported (81). Upon sequence comparison, our newfound gene proved to be equivalent to *pilA2*, which, in the 130b genome (14), is a monocistronic open reading frame (ORF) ABXK18_07095. RNA-seq identified another putative pilin gene that had not been reported before, although this gene was not differently expressed in the *cas2* mutant (90). This monocistronic ORF (ABXK18_07100) was positioned 196 bp after *pilA2* and was equivalent to *pilA1* of strain Paris (81). The *pilA2-pilA1* locus was located apart from all previously defined T4P-associated genes (Fig. 1A). In line with RNA-seq (90), qRT-PCR confirmed that *pilA2* and *pilE* are less expressed in the *cas2* mutant (Fig. 1B).

BLASTP detected *pilA2* and *pilA1* in all 55 other *L. pneumophila* strains examined (Table S1), indicating that the genes are conserved in *L. pneumophila*. Besides *L. pneumophila*, there are >65 species in the *Legionella* genus (91). Further searches revealed the presence of *pilA2*/*pilA1* homologs in all *Legionella* species (Fig. 1C), except for *Legionella polyplacis*, a louse symbiont that has a reduced genome (92). Although most species had a single homolog, 15, like *L. pneumophila*, had 2 major pilin homologs, 7 had 3, and 1 (*Legionella tauriniensis*) had 4 (Fig. 1C). Proteins related to PilA2/PilA1 were also encoded by strains representing most other genera in the Legionellales or other deep-branching intracellular γ-proteobacteria (DIG) (Fig. 1C) (93). These proteins included one homolog from *Coxiella burnetii*, the agent of Q fever, and two from *Francisella tularensis*, the agent of tularemia (94, 95). BLASTP searches beyond the DIG indicated that PilA2 (and by extension PilA1, see below) is related to many putative pilins from an array of other bacteria (Table S2A) and known pilins from well-studied T4aP-expressing bacteria (Table S2B).

The 136-amino acid (aa) PilA2 shares ~72% aa identity with the 137-aa PilA1, with their sequence differences occurring along the length of the proteins (Fig. S1A). Analysis using AlphaFold-3 yielded high-confidence models for the structures of PilA2 and PilA1 (Fig. 1D), showing the *N*-terminal α-helices (α1-N and α1-C), αβ-loops, and variable D-regions that are typical of pilins (36, 96, 97). When we aligned these predicted structures, the relatedness between PilA2 and PilA1 was confirmed (Fig. 1D). Further alignments affirmed that PilA2 and PilA1 are related to major pilins from other T4P-expressing bacteria (Fig. 1E, S1B). The relatedness of PilA2 (and PilA1) to major pilins was greater than that of PilE to the same proteins (Fig. S1C), supporting the prior experimental evidence that PilA2 (and perhaps PilA1) is a major pilin of *L. pneumophila*, whereas PilE is a minor pilin (81).

### PilA2 forms competence-associated T4P

**Fig 2:**
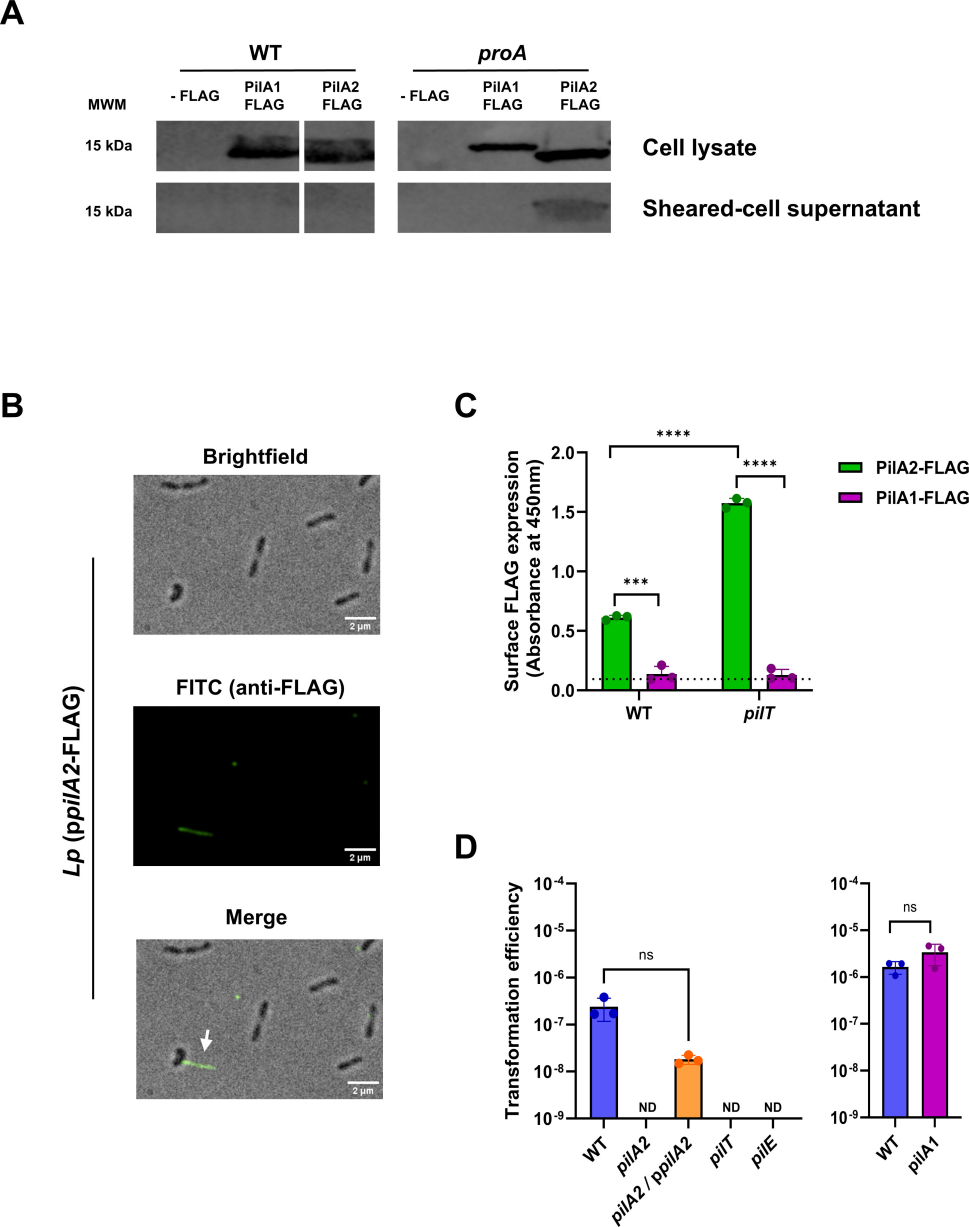
Expression of PilA1, PilA2, and PilA2-containing T4P. (**A**) Strain 130b (WT) and its *proA* mutant AA200 (*proA*) carrying (on a plasmid) IPTG-inducible genes that encode either PilA1 or PilA2 with a *C*-terminal FLAG tag (PilA1-FLAG, PilA2-FLAG) or not encoding a tagged protein (-FLAG) were grown to early stationary phase in BYE broth. Cell lysates (top panels) and sheared-cell supernatants (bottom panels) were subjected to immunoblot using anti-FLAG antibodies. The (only) portions of the blots having proteins recognized by the antibodies, alongside the position of pre-stained molecular weight markers (MWM) in kDa, are presented. For the left panels, the images of the PilA1-FLAG and PilA2-FLAG samples were from non-adjacent lanes, as the white spaces denote. (**B**) *proA* mutant bacteria encoding PilA2-FLAG (*Lp* p*pilA2*-FLAG) were incubated with anti-FLAG antibody and then examined by IF. A representative image of the treated bacteria seen with brightfield or epifluorescence microscopy is shown. In the merged image, the white arrowhead points to a bacterial cell with a long PilA2-containing pilus emanating from its surface. (**C**) WT (WT) and *pilT* mutant (*pilT*) expressing either PilA2-FLAG or PilA1-FLAG or not encoding a FLAG-tagged protein were subjected to whole cell ELISA (three technical replicates/strain), where the presence of FLAG-tagged protein was detected by anti-FLAG antibody-associated absorbance at 450 nm. Data are presented as the means and standard deviations for the absorbance readings. The dashed line denotes the background level of absorbance associated with WT lacking FLAG. Asterisks indicate either the greater surface expression of the FLAG-tag in the bacteria encoding PilA2-FLAG relative to the bacteria encoding PilA1-FLAG or the greater surface expression of the PilA2-FLAG in the *pilT* mutant relative to WT: ***, *P* < 0.001; ****, *P* < 0.0001. (**D**) Strain 130b (WT), *pilA2* mutant NU494 (*pilA2*), *pilA2* mutant NU494 carrying p*pilA2* (*pilA2*/p*pilA2*), *pilT* mutant NU498 (*pilT*), *pilE* mutant NU496 (*pilE*), and *pilA1* mutant NU497 (*pilA1*) were incubated with plasmid DNA, and then, their transformation efficiency was determined. Data are presented as the means and standard deviations from three technical replicates per strain and are representative of the results from three independent experiments. ND denotes those cases where no Kn-resistant colonies were detected, whereas ns denotes non-significant differences between strains. For panels **A–D**, the results presented are representative of the outcome of three independent experiments, except for WT lysates being examined twice.

To begin experimental assessment of PilA2 and PilA1, we introduced plasmids encoding FLAG-tagged versions of PilA2 or PilA1 into WT 130b and its *proA* mutant. The mutant lacks a secreted protease that often degrades other secreted or surface proteins during *in vitro* culture (11, 14), and hence, we reasoned its usage might ease extracellular detection of the pilins. Immunoblot analysis of cell lysates confirmed that PilA1-FLAG and PilA2-FLAG are expressed when WT and *proA* mutant are standardly grown at 37°C in BYE broth (Fig. 2A, top). When we examined supernatants obtained after shearing suspensions of the *proA* mutant, PilA2-FLAG was detected (Fig. 2A, bottom), indicating that PilA2 is secreted and likely assembled into pili. IF microscopy confirmed the presence of PilA2 along the length of pili emanating from the surface of the *proA* mutant (Fig. 2B). That PilA2 appeared on pili visible by IF was also evident if FLAG-tagged PilA2 was expressed in WT from *pilA2*’s chromosomal locus (Fig. S2A). Approximately 2% of cells in our samples displayed these long PilA2-containing pili, which is similar to the 10% of 130b bacteria showing long pili as previously visualized by EM (75). To assess the location of PilA2 by another method, we adapted whole-cell ELISA and again found PilA2 on the bacterial surface (Fig. 2C). In T4P-expressing bacteria, PilT promotes pilus retraction, and *pilT* mutants express more pili on their surface and/or extra-long pili (31–35). Since *pilT* occurs in *L. pneumophila* (Fig. 1A) (81), we tested a *pilT* mutant of strain 130b. As determined by ELISA (Fig. 2C) and IF microscopy (Fig. S2B), the mutant had increased levels of PilA2 on its surface and more pili, affirming that PilA2-containing pili are controlled by factors typically associated with T4P. A *pilA2* mutant, but not its complement, was impaired for transformation (Fig. 2D), indicating that PilA2 facilitates DNA uptake by strain 130b, as it does for strains HL-0709-3014 and Paris (81). The *pilT* mutant as well as a *pilE* mutant were also impaired for DNA uptake (Fig. 2D), as expected (81, 82, 86). Together, these data indicated that PilA2 is a major pilin for strain 130b and that PilA2-containing T4P are linked to competence (81). With assays now in hand, we examined, for the first time, the surface location of PilA1. Unlike PilA2, PilA1 was not seen by immunoblotting of sheared cell suspensions, IF microscopy, or ELISA, even when we used a *proA* mutant or *pilT* mutant background in some of these assays (Fig. 2A and C; S2C ). Also, a 130b *pilA1* mutant was not impaired for DNA transformation (Fig. 2D), as was a Paris *pilA1* mutant (81). Thus, under standard conditions, PilA1 is not significantly featured on the *L. pneumophila* surface, and its identity as a functional pilin, major or otherwise, remains obscure. One possible explanation for the lack of surface PilA1 might be that PilA1 prepilin is not cleaved and processed, as suggested by its slower migration during SDS-PAGE relative to PilA2 (Fig. 2A).

### PilA2 majorly promotes *L. pneumophila* growth in low-iron conditions

**Fig 3:**
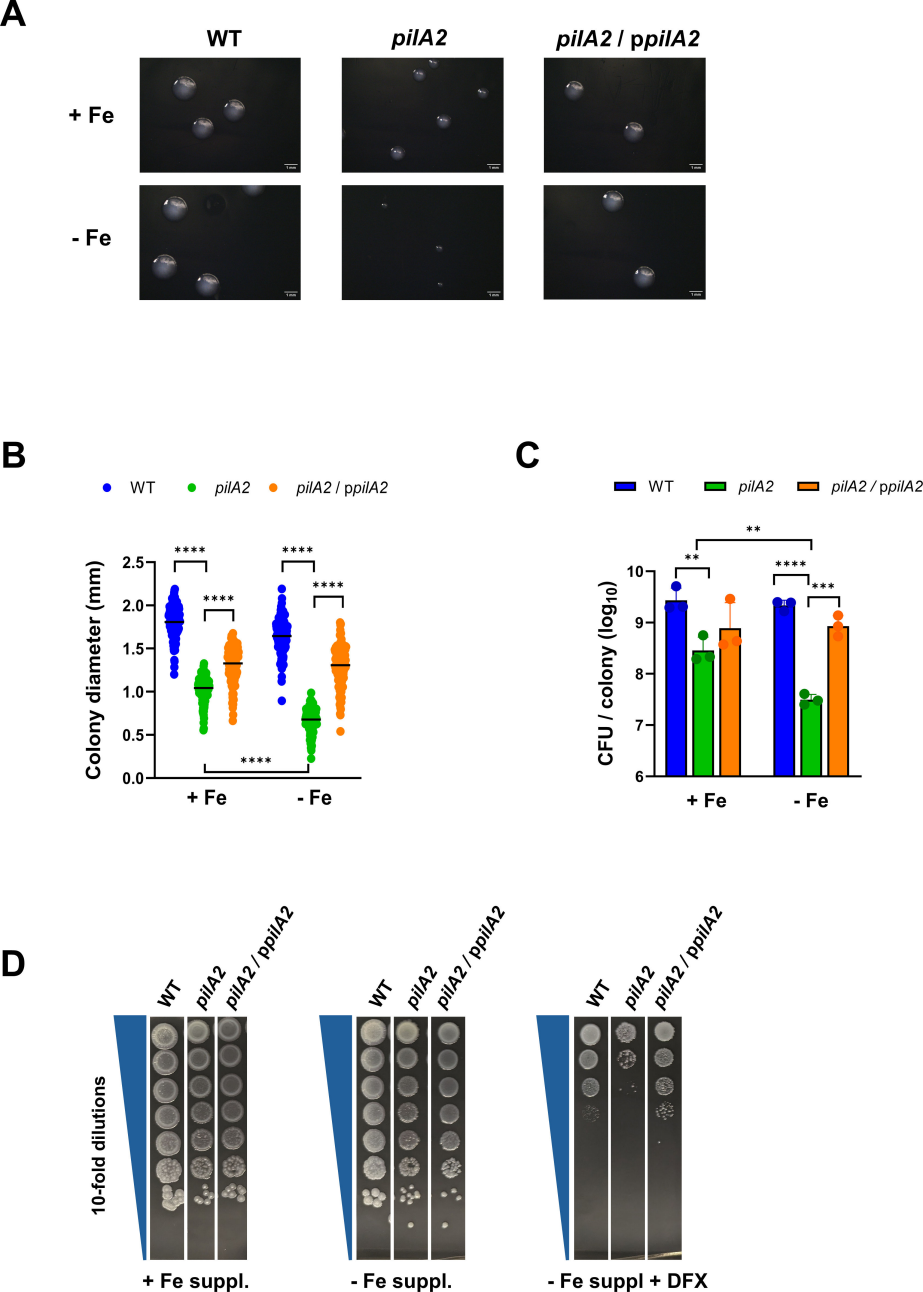
Effect of PilA2 on *L. pneumophila* colony size and efficiency of plating on BCYE agar containing varying amounts of available iron. (**A–C**) WT 130b, *pilA2* mutant NU494 (*pilA2*), and NU494 carrying p*pilA2* (*pilA2* / p*pilA2*) were grown for 3 days on either BCYE agar containing its standard ferric pyrophosphate supplement (+Fe) or lacking that supplement (−Fe), and then, either images of representative colonies were obtained (**A**), the diameters of colonies were measured (**B**), or the numbers of CFU per colony were determined (**C**). In panel **B**, pooled data are presented as the means and standard deviations obtained after examining 150 colonies across three separate experiments, and asterisks indicate the differences in colony size between the *pilA2* mutant vs. WT and complement, as well as the difference in colony size between the mutant grown on BCYE +Fe agar vs. the mutant grown on BCYE −Fe agar: ****, *P* < 0.0001. In panel **C**, pooled data are presented as the means and standard deviations obtained after examining three colonies in each of the three independent experiments done, and the asterisks indicate the differences in CFU/colony between the *pilA2* mutant vs. WT or complement as well as the difference in CFU/colony between the mutant grown on BCYE +Fe agar vs the mutant grown on BCYE −Fe agar: **, *P* < 0.01; ***, *P* < 0.001; ****, *P* < 0.0001. (**D**) WT strain 130b (WT), *pilA2* mutant NU494 (*pilA2*), and NU494 carrying p*pilA2* (*pilA2*/p*pilA2*) were spotted onto BCYE agar that either contained its standard supplement of ferric pyrophosphate (+ Fe suppl.), lacked that iron supplement (− Fe suppl.), lacked that supplement and instead had 8 μM iron chelator DFX added (− Fe suppl. + DFX). Following incubation for 8 days at 37°C, images were taken of the areas of bacterial growth. Comparable results were obtained in more than three independent experiments, including those appearing in later Figures.

After incubating for 3 days on standard BCYE agar at 37°C, the 130b *pilA2* mutant exhibited smaller colonies than WT or the complemented *pilA2* mutant did (Fig. 3A), and this was confirmed when we measured the sizes of the colonies (Fig. 3B). When the agar plates were incubated longer, the mutant’s colonies eventually approximated those of WT; for example, its colonies at day 5 appeared to match the size of WT colonies at day 4 (Fig. S3A). Interestingly, when strains were incubated on BCYE agar that lacked its customary iron supplement, 330 μM ferric pyrophosphate (98), the mutant colonies were even smaller, and the colony size differences between the mutant vs. WT and complement increased (Fig. 3A and B).

These data suggested that PilA2 promotes *L. pneumophila* growth, especially as iron levels in the medium are diminished. To distinguish this possibility from the alternative that the *pilA2* mutant “packs” more tightly within colonies, WT, mutant, and complement colonies were resuspended, vortexed, and plated for CFU. After being harvested from BCYE agar, the mutant had ~10-fold fewer CFU/colony than WT did (Fig. 3C). When bacteria had been grown on non-iron-supplemented agar, the difference between WT and mutant (but not its complement) increased to ~70-fold (Fig. 3C), further indicating that PilA2 promotes the growth of 130b in lower-iron conditions. As an alternate way of assessing strain differences, re-suspended bacteria were subjected to serial dilution, and aliquots from the dilutions were spotted onto either BCYE agar, BCYE agar lacking added iron, or BCYE agar lacking the iron supplement but having added deferoxamine (DFX), a Fe^3+^ chelator. All strains showed a similar efficiency of plating (eop) on BCYE agar and BCYE agar lacking added iron (Fig. 3D). However, on the media containing DFX, the eop of the *pilA2* mutant, but not its complement, was impaired by ~100-fold or more (Fig. 3D and see Fig. 5). An independently made *pilA2* mutant of strain 130b also had impaired growth on low-iron BCYE agar (Fig. S3B). Moreover, impaired growth was evident when we examined a *pilA2* mutant of strain Togus-1 (Fig. S3C and D), indicating that our initial observations were not oddly specific to strain 130b.

**Fig 4:**
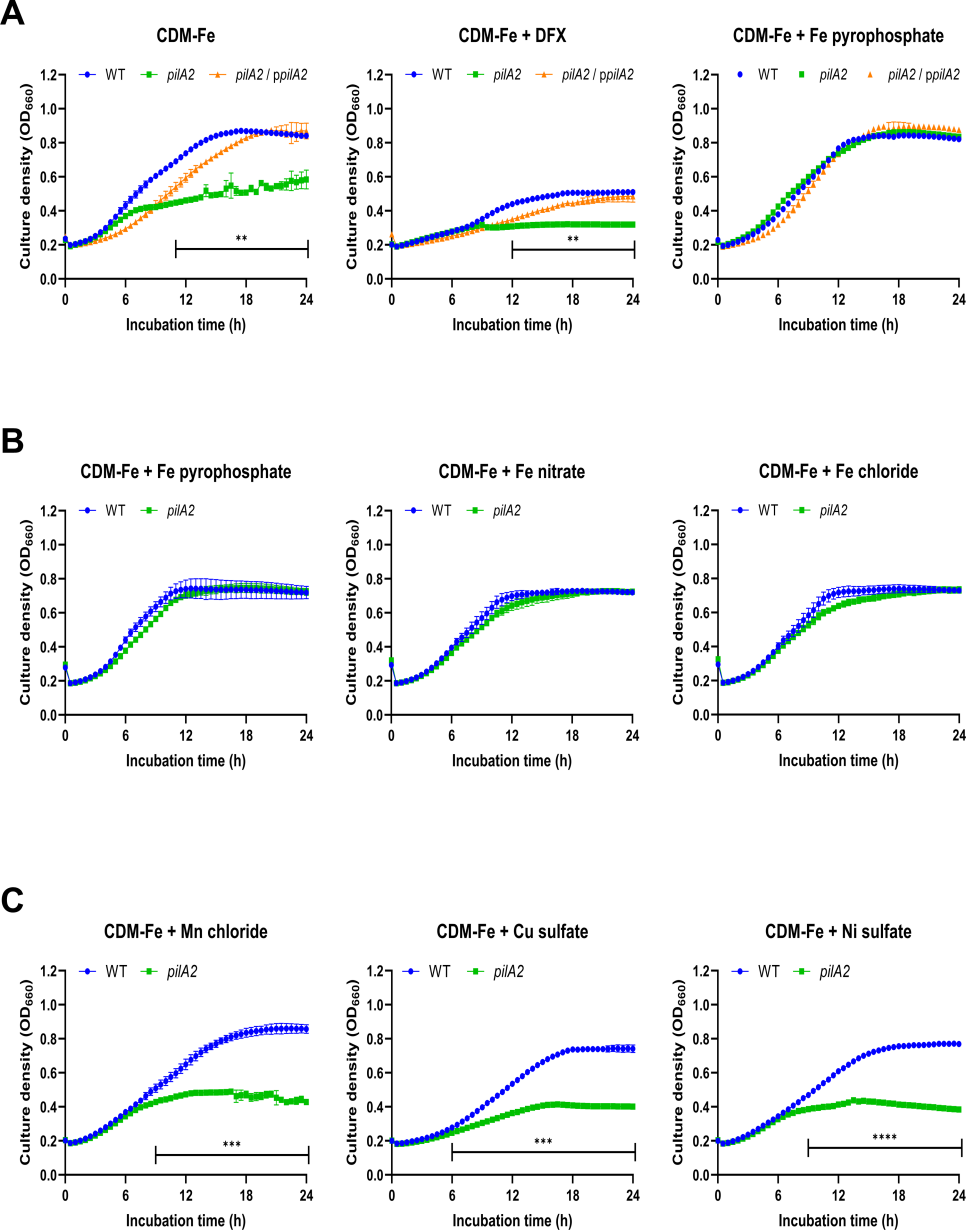
Effect of PilA2 on *L. pneumophila* growth in CDM broth containing varying amounts and types of iron. (**A**) WT 130b (WT), *pilA2* mutant NU494 (*pilA2*), and NU494 carrying p*pilA2* (*pilA2*/p*pilA2*) were inoculated into either CDM-Fe broth (CDM-Fe), CDM-Fe broth with 2 μM DFX (CDM-Fe, +DFX), or CDM-Fe broth with 5 μM ferric pyrophosphate (CDM-Fe, + Fe pyrophosphate) within a microtiter plate. Upon incubation at 37°C, bacterial growth was monitored by OD_660_ readings taken every hour. Data are presented as the means and standard deviations from three technical replicates. In CDM-Fe broth, the *pilA2* mutant grew differently from WT and the complemented mutant at *t* = 11 h and beyond, **P < 0.01. In the CDM-Fe +DFX broth, the *pilA2* mutant grew unlike WT and the complemented mutant at *t* = 12 h and beyond, **P < 0.01. (**B**) Following the steps outlined in panel **A**, WT 130b (WT) and *pilA2* mutant NU494 (*pilA2*) were monitored for growth in CDM-Fe broth with either 5 μM ferric pyrophosphate added (CDM-Fe, + Fe pyrophosphate), 5 μM ferric nitrate added (CDM-Fe, + Fe nitrate), or 5 μM ferric chloride added (CDM-Fe, + Fe chloride). (**C**) Following the steps outlined in panel **A**, WT 130b and *pilA2* mutant NU494 (*pilA2*) were monitored for growth in CDM-Fe broth with either 5 μM manganese chloride added (CDM-Fe, + Mn chloride), 5 μM cupric sulfate added (CDM-Fe, + Cu sulfate), or 5 μM nickel sulfate added (CDM-Fe, + Ni sulfate). In the Mn-supplemented broth, the *pilA2* mutant grew differently from WT at *t* = 9 h and beyond, ***P < 0.001. In the Cu-supplemented broth, the *pilA2* mutant grew differently from WT at *t* = 6 h and beyond, ***P < 0.001. In the Ni-supplemented broth, the *pilA2* mutant grew differently from WT at *t* = 9 h and beyond, ****P < 0.0001. For panel **A–C**, the data presented are representative of the results from three independent experiments.

To determine if the *pilA2* mutants’ impaired ability to grow under low-iron conditions was peculiar to growth on a solid medium or on BCYE-based agar specifically, we tested the 130b *pilA2* mutant for its ability to grow in a chemically defined liquid medium that lacked added iron (CDM-Fe broth). This broth consists of 20 aa (with cystine replacing cysteine), α-ketoglutarate, glutathione, pyruvate, and nine trace metals other than iron, KH_2_PO_4_, NaCl, and MOPS buffer. Based on inductively coupled plasma mass spectrometry, the background level of iron in CDM-Fe broth is ~0.14 µM (14). Over the course of a 24-h experiment, the *pilA2* mutant, but not its complement, exhibited markedly impaired growth in CDM-Fe broth, with there being little if any growth after ~6 h (Fig. 4A). Moreover, the mutant’s growth was essentially abolished by the addition of DFX into the medium (Fig. 4A). However, the mutant’s growth could be rescued by the addition of Fe^3+^ pyrophosphate into the CDM-Fe broth (Fig. 4A). This growth-restoration also occurred when Fe^3+^ nitrate or Fe^3+^ chloride was added (Fig. 4B). Restoration did not occur when manganese, copper, or nickel (i.e., metals that may substitute for iron as a co-factor) were added (Fig. 4C). Overall, these data indicated that PilA2 promotes *L. pneumophila* growth in low iron on both surfaces and in liquid and in media rich in various other nutrients (BCYE) and media that only contain aa, salts, and other trace metals (CDM). Since iron assimilation is a key factor in *L. pneumophila* persistence in water and during infection (99, 100), we sought to better understand the effect of PilA2 on growth in low iron.

### Other components of the T4P apparatus are not required for optimal growth of *L. pneumophila* on low-iron media

**Fig 5:**
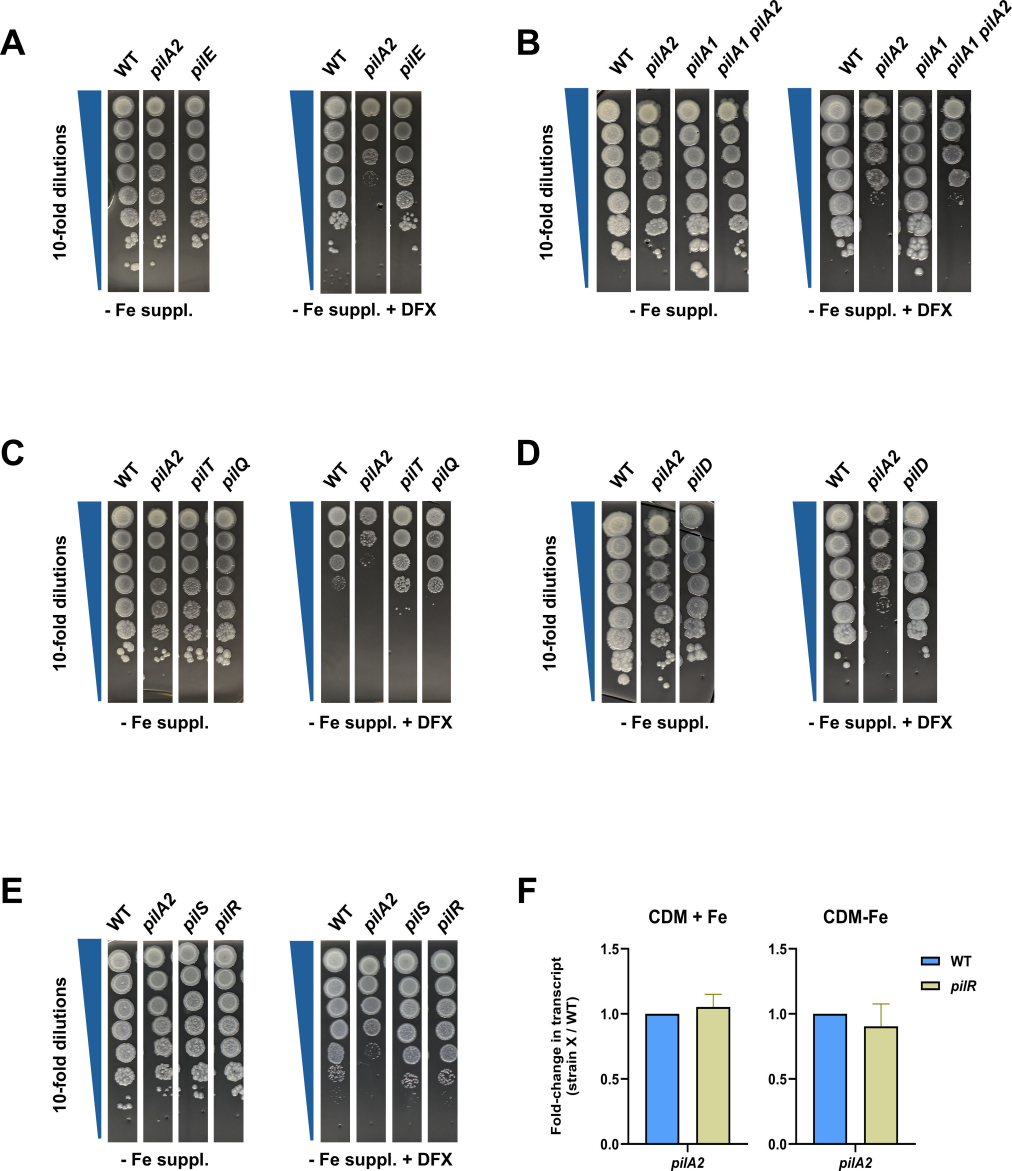
Effects of other T4P-related genes on *L. pneumophila* growth on BCYE agar containing varying amounts of iron. (**A–E**) WT strain 130b (WT), the *pilA2* mutant NU494 (*pilA2*), and either the *pilE* mutant NU496 (*pilE*) (**A**), *pilA1* mutant NU497 (*pilA1*) and *pilA1 pilA2* mutant NU503 (*pilA1 pilA2*) (**B**), *pilT* mutant NU498 (*pilT*) and *pilQ* mutant NU279 (*pilQ*) (**C**), *pilD* mutant NU272 (*pilD*) (**D**), or *pilS* mutant NU500 (*pilS*) and *pilR* mutant NU501 (*pilR*) (**E**) were tested for eop on BCYE agar that lacked its iron supplement (− Fe suppl.) or BCYE agar that lacked the iron supplement but had 8–10 μM DFX added (− Fe suppl. + DFX). For panels **A–E**, the images presented are representative of the results from three independent experiments. (**F**) WT and *pilR* mutant NU501 were grown in CDM broth supplemented with ferric pyrophosphate (CDM+Fe) or CDM broth lacking added iron (CDM-Fe), and then, the levels for *pilA2* mRNA were determined using RNA from three biological replicates (i.e., three independent trials), each in technical triplicate. Data are presented as the means and standard deviations for the fold change in the mutant’s transcript levels, beside WT values set to 1.0.

In contrast to the *pilA2* mutant, the *pilE* mutant did not have a reduced eop on DFX-containing media (Fig. 5A). The *pilA1* mutant also exhibited a normal growth pattern (Fig. 5B), and a mutant lacking *pilA1* and *pilA2* was no more impaired than the *pilA2* mutant was(Fig. 5B). Normal growth for the *pilE* mutant and *pilA1* mutant was also observed when we tested them in CDM-Fe broth (Fig. S4A). These findings indicated that *pilA2,* but not all pilin genes, are needed for full growth on low iron. Mutants lacking the PilQ secretin (77) or PilT ATPase (above) were also not impaired for eop on low-iron BCYE agar and for growth in CDM-Fe broth (Fig. 5C and Fig. S4B), implying that PilA2’s role is not linked to its placement into and retraction along with T4P or that *L. pneumophila* growth on low iron requires T4P in a general sense. To test if the mature form of PilA2 is necessary for optimal growth, we tested a mutant that lacks PilD, the inner-membrane peptidase that cleaves the *N*-termini from pre-pilins and then methylates the processed pilins, as a prerequisite to their polymerization into filaments (77). The *pilD* mutant was not impaired for eop on low-iron BCYE agar (Fig. 5D). Moreover, it, unlike the *pilA2* mutant, was not impaired during the replicative phase in CDM-Fe broth cultures (Fig. S4B), although the *pilD* mutant did exhibit impaired survival later in the stationary phase. Together, these data suggest that a pre-pilin form of PilA2 facilitates growth in low iron. Besides processing pre-pilins for T4P, PilD also cleaves pre-pilin-like proteins that are part of the T2SS (77, 101). Thus, the fact that the *pilD* mutant did not mimic the *pilA2* mutant on the low-iron media also meant that PilA2’s effect was not due to a possible inclusion of PilA2 into the T2SS apparatus. Incidentally, the *pilT* mutant and *pilQ* mutant, but not the *pilD* mutant, appeared to grow slightly better than WT did on the low-iron BCYE agar (Fig. 5C and D), although the reason for this remains unclear.

In some T4P-expressing bacteria, unprocessed major pilins in the inner membrane interact with the PilS/PilR two-component signaling pathway, where PilS is a transmembrane-sensor and PilR is its cytoplasmic partner that modulates transcription of the pilin gene and other types of genes (102–108). In *Geobacter sulfurreducens*, the PilS/PilR pathway impacts the reduction of soluble and insoluble Fe^3+^ (109, 110), suggesting that a link between PilA2 and PilS/PilR might explain our results. Examination of the *L. pneumophila* genome revealed a previously unreported operon (Fig. 1A) whose first ORF (i.e., ABXK18_10615 in strain 130b) is predicted to encode a protein with similarity to known PilS proteins (e.g., 36% aa identity with *P. aeruginosa* PilS [*E* = 1e−80] and 26% aa identity with *G. sulfurreducens* PilS [*E* = 8e−40]) and whose second ORF (i.e., ABXK18_10610 in strain 130b) is predicted to encode a homolog of PilR proteins (e.g., 60% aa identity with *P. aeruginosa* PilR [*E* = 0] and 43% aa identity with *G. sulfurreducens* PilR [*E* = 9e−127]) (103, 109, 111). However, neither a *pilS* mutant of strain 130b nor a 130b *pilR* mutant had a reduced eop on low-iron BCYE agar or impaired growth in CDM-Fe broth (Fig. 4E and Fig. S4C), indicating that the PilS/PilR pathway does not explain the PilA2’s effect on *L. pneumophila* growth in low iron. Incidentally, when grown under low-iron conditions, the *pilR* mutant had slightly lower but not statistically different levels of *pilA2* mRNA (Fig. 4F), suggesting that a total loss of *pilA2* expression vs. slight reductions in expression is needed to observe the impaired growth phenotype. Overall, these experiments indicated that PilA2’s role in promoting *L. pneumophila* growth in low iron does not require the protein’s inclusion in T4P, formation of mature pilin in the periplasm, or engagement with the PilS/PilR pathway at the inner membrane. Thus, we posited that a heretofore undescribed aspect of the major pilin is enhancing growth in low iron.

### Lack of functional overlap between PilA2 and other mediators of *L. pneumophila* growth in low iron

**Fig 6:**
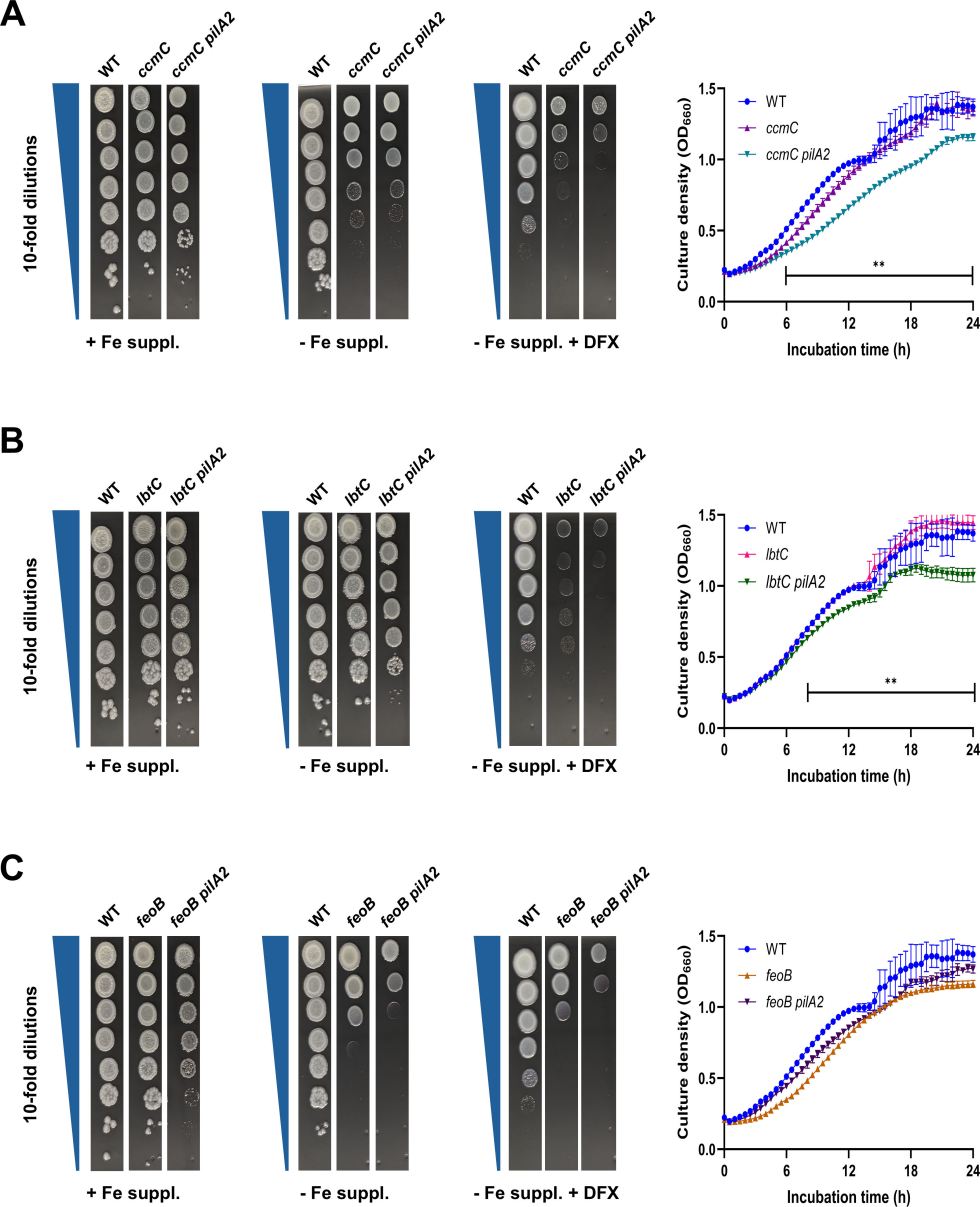
Effect of PilA2 on *L. pneumophila* growth on low-iron BCYE agar in the absence of known mediators of iron acquisition. (**A**) WT strain 130b (WT), *ccmC* mutant NU208 (*ccmC*), and *ccmC pilA2* mutant NU506 (*ccmC pilA2*) were monitored either for eop on BCYE agar that contained its usual iron supplement (+ Fe suppl.), lacked that supplement (− Fe suppl.), or lacked that supplement but had 10 μM iron chelator DFX added (− Fe suppl. + DFX) (three left-most panels) or for growth in microtiter-plate wells (*n* = 4, per strain) containing BYE broth lacking its usual iron supplement (BYE-Fe broth) (right panel). For the experiment in BYE-Fe broth, the growth of the *ccmC pilA2* mutant was different from that of the parental *ccmC* mutant at 6 h and beyond, **, *P* < 0.01. (**B**) WT strain 130b (WT), *lbtC* mutant NU305 (*lbtC*), and *lbtC pilA2* mutant NU504 (*lbtC pilA2*) were monitored for growth on and in low-iron media, as described in panel **A**. For the experiment in BYE-Fe broth, the growth of the *lbtC pilA2* mutant was different from that of the parental *lbtC* mutant at 8 h and beyond, **, *P* < 0.01. (**C**) The abilities of WT strain 130b (WT), *feoB* mutant NU269 (*feoB*), and *feoB pilA2* mutant NU505 (*feoB pilA2*) to grow on or in low-iron media were assessed as outlined in panel **A**. For panels **A–C**, all the strains were tested for growth in BYE-Fe broth at the same time; however, to help the reader more easily visualize the results, we presented the mutant data in the three different graphs, with the WT data being reproduced in each of those graphs. For panels **A–C**, the presented data are representative of the results from three independent experiments.

We have identified pathways that *L. pneumophila* uses to grow optimally in low-iron conditions (14, 99, 112–136). As one pathway, *L. pneumophila* imports Fe^2+^ via its FeoB inner membrane-transporter (118). As a second pathway, the bacterium secretes the Fe^3+^ chelator rhizoferrin (116, 133, 135, 136). This siderophore is made by LbtA and exported via LbtB (123, 133), and Fe^3+^-rhizoferrin is imported by outer membrane LbtU and inner membrane LbtC (128, 130). A third promoter of *L. pneumophila* growth on low-iron media is the cytochrome *c* maturation (*ccm*) locus, which encodes an inner membrane exporter of heme that also incorporates heme into periplasmic apo-cytochromes (119, 120, 129). As a next step to discerning how PilA2 might be promoting growth on low iron, we asked if PilA2 operates on one of the above-mentioned pathways. Thus, we introduced a *pilA2* mutation into previously made 130b mutants that lack either *ccmC*, *lbtC*, or *feoB,* and then tested these double mutants for relative growth in low iron. The *ccmC pilA2* mutant was more impaired than the parental *ccmC* mutant was on both low-iron BCYE agar and in low-iron BYE broth (Fig. 6A), indicating that PilA2 does not function within the Ccm pathway. The *lbtC pilA2* mutant was more impaired than its parental *lbtC* mutant was in both low-iron conditions (Fig. 6B), implying that PilA2 does not operate on the LbtC/rhizoferrin pathway either. Finally, the *feoB pilA2* mutant was more impaired than the parental *feoB* mutant was on low-iron BCYE agar (Fig. 6C), implying that PilA2 and FeoB operate on distinct pathways. However, the *feoB pilA2* mutant was not more impaired than the *feoB* mutant when we tested for growth in low-iron BYE broth (Fig. 6C), suggesting that there might be some overlap between PilA2 and FeoB, depending upon assay conditions. Incidentally, the *feoB* mutant and *ccmC* mutant were impaired (relative to WT) on BCYE-Fe agar but not in BYE-Fe broth (Fig. 6B and C) has been observed before (129, 136). Together, these data raised the possibility that PilA2 functions within an entirely novel pathway, although it might also have a previously undescribed role within the FeoB pathway.

### PilA2 promotes iron levels in *L. pneumophila* cells

**Fig 7:**
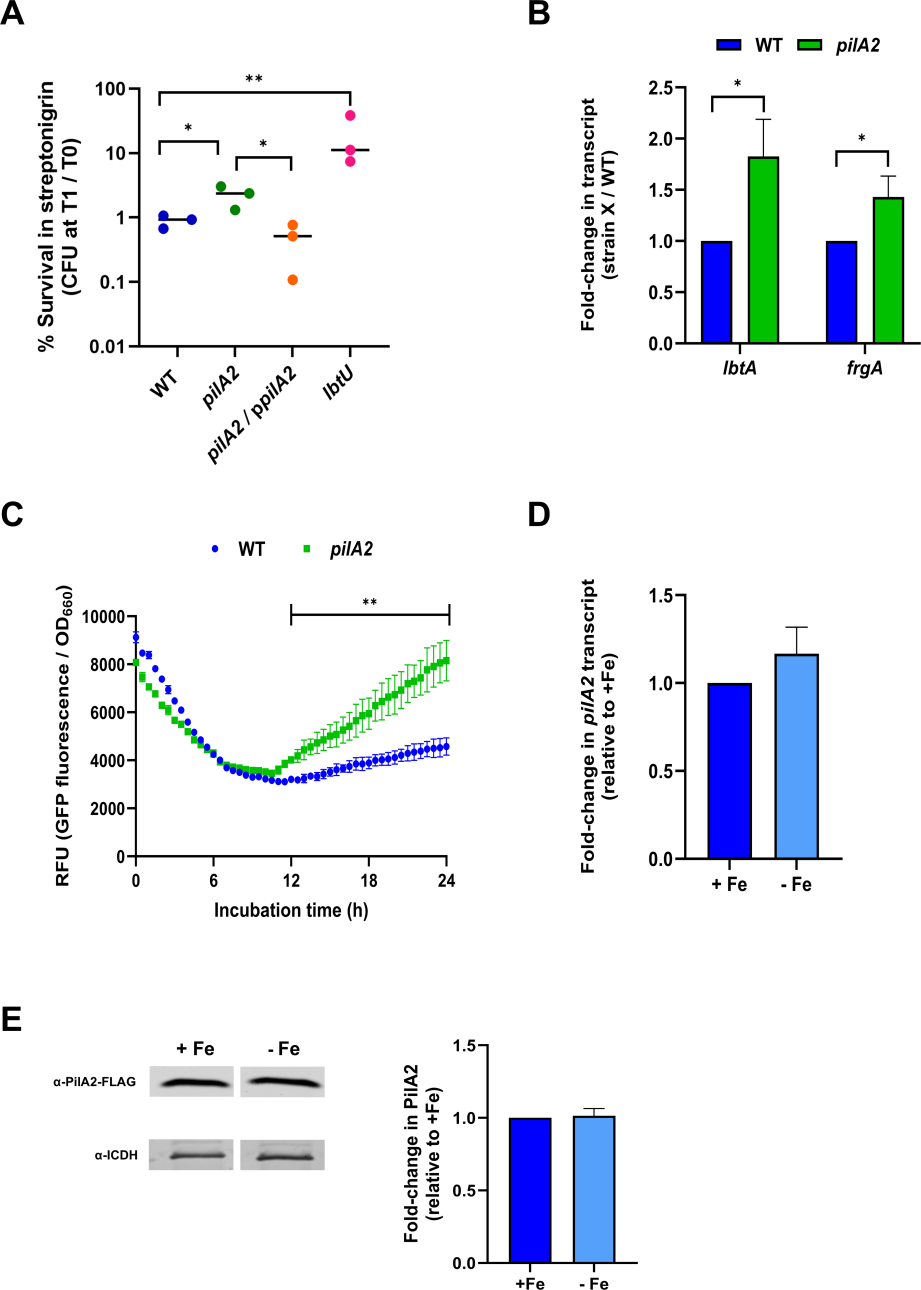
Effect of PilA2 on iron levels within *L. pneumophila* cells, and the effect of low iron on *pilA2* expression. (**A**) WT 130b (WT), *pilA2* mutant NU494 (*pilA2*), NU494 carrying p*pilA2* (*pilA2*/p*pilA2*), and *lbtU* mutant NU383 (*lbtU*) were exposed (in technical triplicate) to streptonigrin for 1 h, and then, the percent survival was calculated. The data presented are the values pooled from three independent experiments. Asterisks indicate differences in streptonigrin-resistance between the *pilA2* mutant vs. WT and complement as well as the difference between WT and the *lbtU* mutant: *, *P* < 0.05; **, *P* < 0.01. (**B**) WT and *pilA2* mutants were grown in CDM-Fe broth, and then, the mRNA levels for *lbtA* and *frgA* were determined by qRT-PCR using RNA from three biological replicates (i.e., three independent experiments), each done in technical triplicate for each strain. Data are presented as the means and standard deviations for the fold changes in the mutant’s transcripts, beside WT values set to 1.0. Asterisks indicate significant increases in both transcript levels for the mutant, relative to WT: *, *P* < 0.05. (**C**) WT and *pilA2* mutant strains expressing GFP under the control of the iron-repressed *frgA* gene were grown in CDM broth containing 5 µM ferric pyrophosphate (*n* = 3, per strain). Data are presented as relative fluorescence units (RFU), which are derived from the ratio of GFP-associated fluorescence levels over the extent of bacterial growth (OD_660_). Asterisks indicate that RFU values were greater for the mutant relative to WT at *t* = 12 h and beyond: **, *P* < 0.01. These data are representative of the results obtained from three independent experiments. (**D**) WT bacteria were inoculated into CDM broth supplemented with ferric pyrophosphate (+Fe) or CDM broth lacking added iron (-Fe), and then, *pilA2* mRNA levels were determined by qRT-PCR using RNA from three biological replicates (i.e., three independent experiments), each done in technical triplicate. Data are presented as the means and standard deviations for the fold change in the mutant’s transcript levels when grown in CDM-Fe broth vs. when grown in CDM+Fe broth, beside WT values set to 1.0. (**E**) WT expressing PilA2-FLAG was grown as in (**D**). Cell lysates were subjected to SDS-PAGE, with equal amounts of protein loaded per sample, and immunoblotted utilizing either anti-FLAG antibodies (left panel, top row) or anti-ICDH antibodies (left panel, bottom row). The (only) portions of the blots having proteins recognized by the antibodies are presented, and these data are representative of the results from three independent experiments. Following densitometry and normalization against the ICDH bands, data are presented as the means and standard deviations for the fold change in PilA2-FLAG expression from bacteria grown in CDM-Fe broth relative to PilA2-FLAG expression from bacteria cultured in CDM+Fe broth, whose values are set to 1.0 (right panel).

As one explanation for the impaired growth of the *pilA2* mutant on low-iron media, we posited that PilA2 promotes iron uptake into the bacterial cell. Thus, we sought to discern if the *pilA2* mutant has lowered levels of cellular iron. First, we tested the mutant’s resistance to streptonigrin, an antibiotic whose toxicity in *L. pneumophila* and other bacteria is increased as cellular iron levels increase (53, 118, 137, 138). As a control in the assay, we used a mutant lacking the LbtU receptor for the rhizoferrin siderophore (128) and found the *lbtU* mutant to be ~10-fold more resistant to streptonigrin than WT 130b was (Fig. 7A). The *pilA2* mutant had ~3-fold increased resistance relative to WT and ~5-fold increased resistance relative to its complement (Fig. 7A), suggesting that it also has lower levels of iron. For confirmation by an alternate means, we grew WT and *pilA2* mutant in CDM broth and used qRT-PCR to compare the levels of expression of *frgA* and *lbtA*, two genes that are known to be induced by low iron (14, 115, 123). The induction of both genes was more pronounced in the *pilA2* mutant (Fig. 7B), further indicating that this strain has lowered levels of available iron. To bolster these results, we monitored the cellular levels of green fluorescent protein (GFP) as expressed from the *frgA* promoter and normalized to the levels of bacterial growth (139–141). Upon inoculation into the CDM broth, the *pilA2* mutant exhibited increased GFP expression relative to WT, and this was co-incident with slowed mutant growth (Fig. 7C). Taken together, these data documented that PilA2 promotes iron uptake into the cell and/or the proper assimilation of the internalized iron, which most likely explains why the protein facilitates bacterial growth in low-iron media. qRT-PCR demonstrated that the levels of *pilA2* mRNA do not increase significantly when WT is grown in CDM-Fe broth vs. in CDM+Fe broth (Fig. 7D), indicating that *pilA2* transcription, unlike that of *frgA* and *lbtA*, is not (highly) regulated by the amount of iron in the growth media, which aligns with the gene’s promoter region not having sequences that match the consensus Fur box of *L. pneumophila* (14). Immunoblot analysis of cell lysates indicated that levels of PilA2 also do not increase when bacteria are grown in the iron-deplete media (Fig. 7E). These results suggest a model in which the role of PilA2 for iron acquisition/assimilation does not entail heightened PilA2 expression but may involve a change in the protein’s interactions and/or location.

### PilA2 impedes *L. pneumophila* biofilm formation, whereas PilA1 promotes biofilm formation

**Fig 8:**
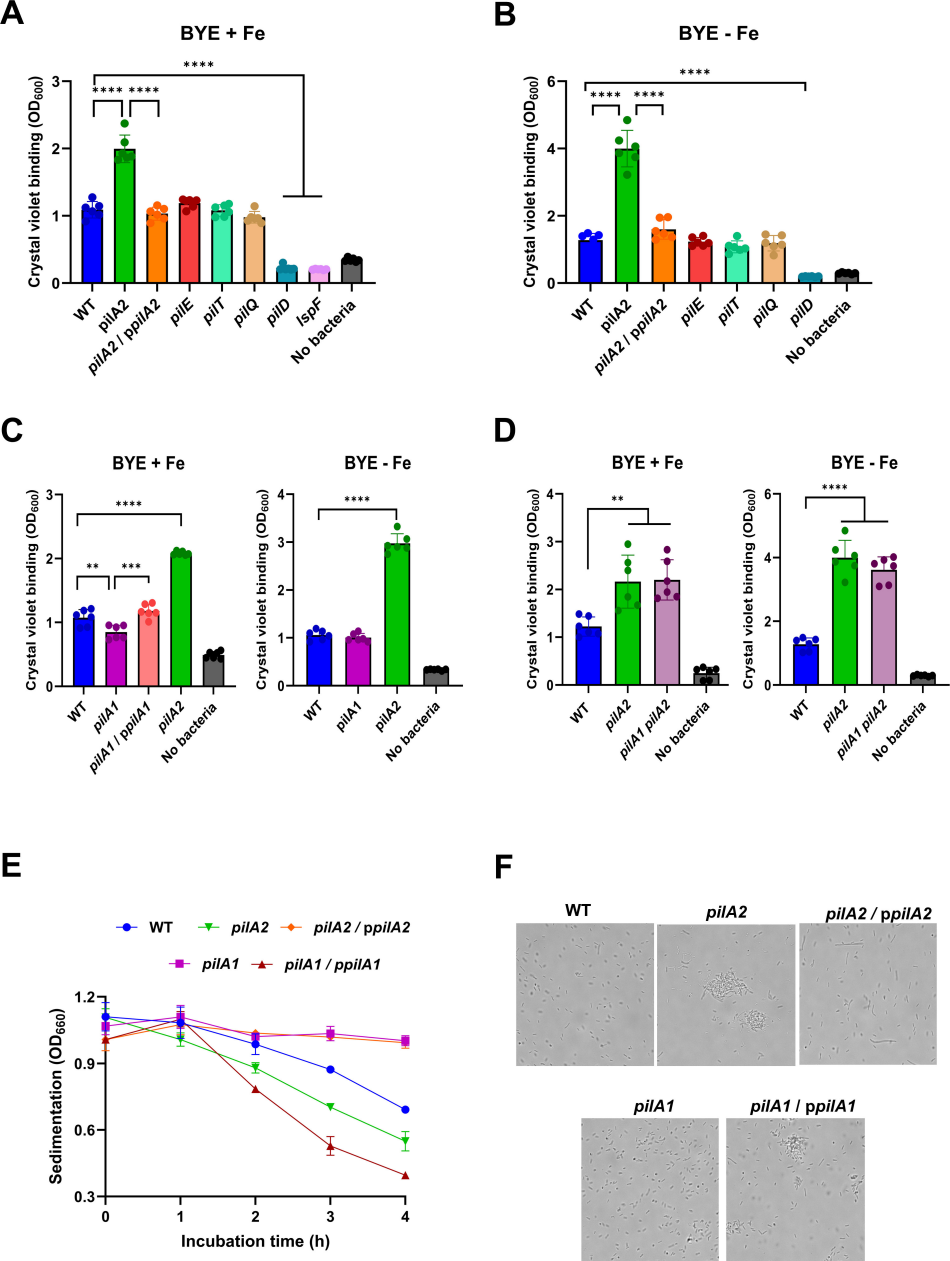
Effect of PilA2 and PilA1 on *L. pneumophila* biofilm formation and autoaggregation. **(A and B**) WT 130b, *pilA2* mutant NU494 (*pilA2*), NU494 carrying p*pilA2* (*pilA2*/p*pilA2*), *pilE* mutant NU496 (*pilE*), *pilT* mutant NU498 (*pilT*), *pilQ* mutant NU279 (*pilQ*), *pilD* mutant NU272 (*pilD*), and *lspF* mutant NU275 (*lspF*) were resuspended in either BYE broth containing its standard amount of added iron (BYE + Fe) (**A**) or BYE broth lacking the iron supplement (BYE – Fe) (**B**) and then, the suspensions were added into the wells of a microtiter plate, and biofilm formation was assessed. Data are presented as the means and standard deviations from six technical replicates. Asterisks indicate the differences in biofilm levels between the *pilA2* mutant vs. WT and complement as well as the differences between WT and the *pilD* mutant and *lspF* mutant: ****, *P* < 0.0001. (**C**) WT 130b, *pilA1* mutant NU497 (*pilA1*), NU497 carrying p*pilA1* (*pilA1*/p*pilA1*), and *pilA2* mutant NU494 (*pilA2*) were assessed for biofilm formation in either BYE +Fe broth (left) or BYE –Fe broth (right), as described in panel **A**. Asterisks indicate differences in biofilm levels between the *pilA1* mutant vs. WT and complement as well as the difference between WT and the *pilA2* mutan: **, *P* < 0.01; ***, *P* < 0.001; ****, *P* < 0.0001. (**D**) WT 130b, *pilA2* mutant NU494 (*pilA2*), and *pilA1 pilA2* mutant NU503 (*pilA1 pilA2*) were assessed for biofilm formation in either BYE +Fe broth (left) or BYE –Fe broth (right), as described in panel **A**. The data contained in the BYE -Fe (right) graph are from the same experiment as the data in panel **B**; however, the data for the double mutant (along with repeat presentations of the data for WT and *pilA2* mutant) were placed here to help facilitate the flow of discussion in the main text. Asterisks indicate the differences in biofilm formation between WT and the *pilA2* mutant and *pilA1 pilA2* mutant: **, *P* < 0.01; ****, *P* < 0.0001. (**E**) For WT strain 130b (WT), *pilA2* mutant NU494 (*pilA2*), *pilA2* mutant NU494 carrying p*pilA2* (*pilA2*/p*pilA2*), *pilA1* mutant NU497 (*pilA1*), and *pilA1* mutant NU497 carrying p*pilA1* (*pilA1*/p*pilA1*) sedimentation was assessed by measuring drops in the OD_660_ of statically incubated cell suspensions. Data are presented as the means and standard deviations from three technical replicates. The two mutants behaved differently from WT at *t* = 3 h and beyond (*P* < 0.001) and from their respective complements at *t* = 2 h and beyond (*P* < 0.001). (**F**) Following static incubation, as noted in panel **E**, strain aggregation was assessed at 4 h by microscopically examining an aliquot taken from the mid-point in the tube. Data in panels **A–F** are representative of the results obtained from three independent experiments.

Since persistence in biofilms is critical for *L. pneumophila* survival, we tested our mutants for biofilm formation, as standardly done (11, 14, 136, 142). The *pilA2* mutant produced approximately twice the amount of biofilm as WT (Fig. 8A), although the two strains had comparable growth in the assay medium, that is, BYE +Fe broth (Fig. S5A). The complemented *pilA2* mutant behaved like the WT did (Fig. 8A), affirming that PilA2 impedes biofilm formation. Unlike the *pilA2* mutant, the *pilE* mutant, *pilT* mutant, and *pilQ* mutant did not show changes in biofilm (Fig. 8A), indicating that (i) T4P are not critical for biofilm formation, at least under these assay conditions, and (ii) the inhibitory effect of PilA2 on biofilm was independent of its assembly into T4P. That a *pilD* mutant was entirely lacking biofilm formation (Fig. 8A) was likely due to the previously noted role of PilD in the T2SS and the documented importance of T2SS substrates in *L. pneumophila* biofilm formation (143, 144). Indeed, a *lspF* mutant behaved similarly to the *pilD* mutant in the biofilm assay (Fig. 8A). Since other phenotypes of the *pilA2* mutant were affected by environmental levels of iron, we assessed biofilm formation utilizing BYE-Fe broth as the assay medium. The *pilA2* mutant, but none of the other strains tested, now produced even more biofilm than WT did (Fig. 8B), although the mutant now showed less planktonic growth in the assay medium (Fig. S5B), as expected. The *pilA2* mutant also exhibited more biofilm when we performed the assays at 30°C (Fig. S6), as done before for *L. pneumophila* strains (11, 14, 136). The *pilA1* mutant uniquely displayed a reduction in biofilm (Fig. 8C). A complemented *pilA1* mutant lacked this phenotype (Fig. 8C), indicating that PilA1 promotes biofilm formation. Although the effect of PilA1 was on the modest side, it nonetheless provided a first clue that PilA1 can be functional and may be part of an adhesive structure. A *pilA1 pilA2* mutant gave a level of biofilm akin to that of a *pilA2* mutant (Fig. 8D), indicating that the increased biofilm shown by the *pilA2* mutant does not involve PilA1.

Prompted by the biofilm results, we tested the role of *pilA2* and the other T4P genes in auto-aggregation. In non-agitated liquid, *L. pneumophila* strains gradually aggregate and settle to the bottom of the tube, reducing the suspension’s optical density (OD) (11, 14, 145–147). The *pilA2* mutant, but not its complement, sedimented more rapidly than WT did (Fig. 8E), suggesting that the strain is hyperaggregative. When the bacteria were resuspended by vortexing, the mutant’s OD matched that of WT, indicating that the mutant’s phenotype was not due to cell lysis. Moreover, under light microscopy, the *pilA2* mutant, unlike WT and complement, was more aggregated (Fig. 8F). In contrast, the *pilA1* mutant exhibited no sedimentation, but its complement sedimented most rapidly and showed aggregation (Fig. 8E and F). Together, these results suggested that PilA1 and perhaps some form of PilA1-containing structure (which we have not yet been able to visualize) aids in *L. pneumophila* auto-aggregation, whereas the expression of PilA2 impedes those interactions. Overall, the mutants’ behaviors in the aggregation assay aligned with their phenotypes in the biofilm assay and suggest that PilA2 and PilA1 may, in some instances, have opposing effects.

### PilA2 and PilA1 are not required for *L. pneumophila* growth in *Acanthamoeba castellanii*

**Fig 9:**
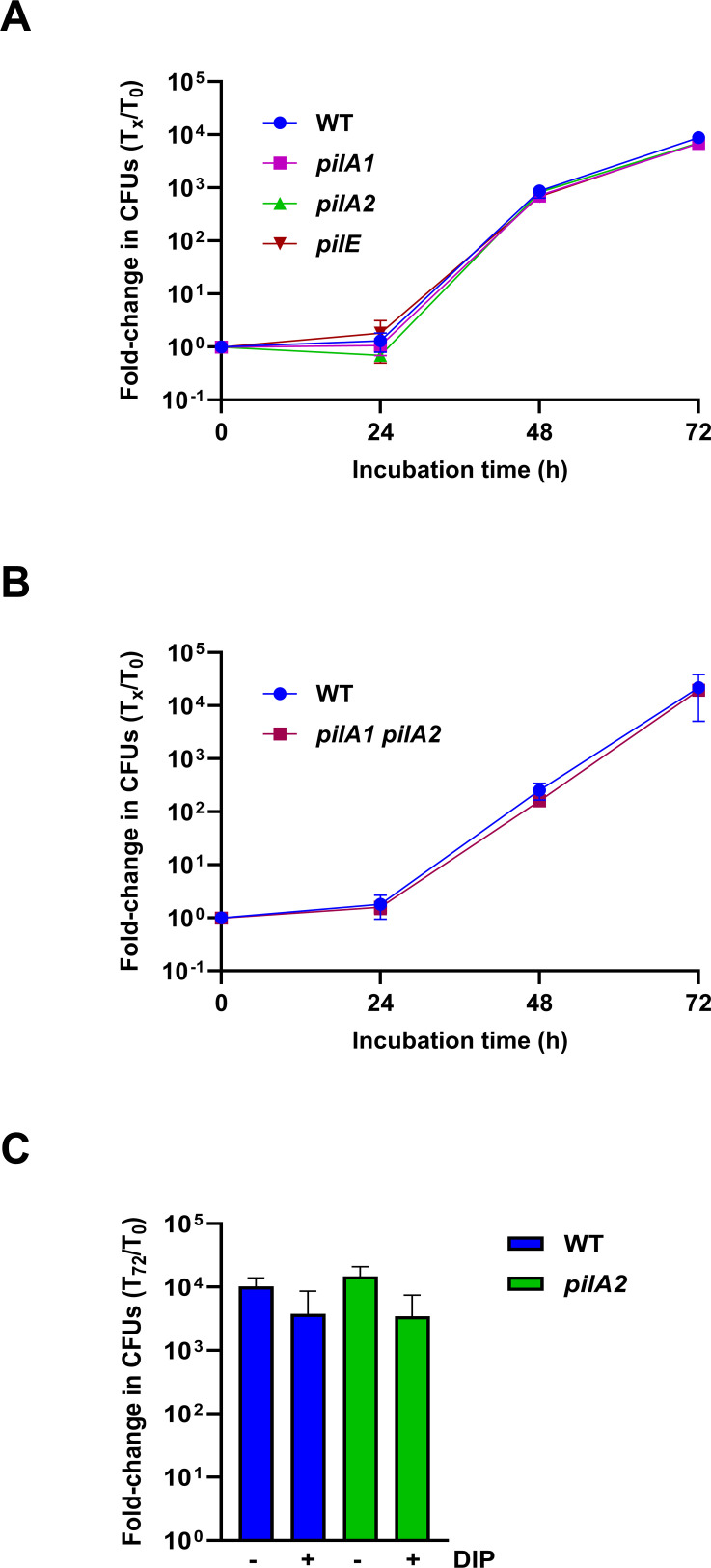
Growth of WT and pilin mutant *L. pneumophila* in *A. castellanii***.** (**A**) *A. castellanii* was infected with strain 130b (WT), *pilA2* mutant NU494 (*pilA2*), *pilE* mutant NU496 (*pilE*), or *pilA1* mutant NU497 (*pilA1*), and then at *t* = 0, 24, 48, and 72 h post-inoculation, CFUs within the co-cultures were determined. The values presented are the means and standard deviations from three technical replicates, and these data are representative of the results obtained from three independent experiments. (**B**) *A. castellanii* monolayers were infected with WT or *pilA1 pilA2* mutant NU503 (*pilA1 pilA2*), and subsequent bacterial growth was recorded, as described in panel **A**. Values presented are the means and standard deviations from three technical replicates, and these data are representative of the results obtained from three independent experiments. (**C**) *A. castellanii* monolayers were infected with WT or *pilA2* mutant bacteria either in the presence or absence of 25 µm DIP, and then, at 72 h post-inoculation, the extent of bacterial growth was determined as described in panel **A**. The values presented are the pooled results from three independent experiments, each done in technical triplicate.

Although the minor pilin PilE is not needed for *L. pneumophila* replication within *A. castellanii* and other amoebae (75, 148), we considered the possibility that the major pilin PilA2 might be important, especially since the study of *pilA2* in strain Paris did not include infection assays (81). However, when co-cultured with *A. castellanii*, the 130b *pilA2* mutant grew to the same extent as WT, as did a *pilE* mutant and *pilA1* mutant (Fig. 9A). To test for a possible functional redundancy between PilA2 and PilA1 during intracellular infection, we tested the *pilA1 pilA2* mutant, but it too was not impaired (Fig. 9B). Finally, given how PilA2’s importance can vary depending on the levels of iron in the growth environment (above), we added the iron chelator dipyridyl (DIP) into the assay medium at a level that does not impact amoebal viability (118, 123). However, the *pilA2* mutant and WT still behaved alike (Fig. 9C). Thus, it appears that neither PilA2 nor PilA1 is required for *L. pneumophila* growth within *A. castellanii*.

## DISCUSSION

Utilizing immunoblot analysis, IF microscopy, and whole-cell ELISA, we documented PilA2 as a major pilin for *L. pneumophila* 130b T4P, including, at least, those pili that are linked to DNA transformation. Combining this finding with a similar result obtained from studying strain Paris (81) and our *in silico* analysis detecting *pilA2* in all *L. pneumophila* strains, we posit that PilA2 is a major pilin across the species. We characterized PilA1 as a close homolog of PilA2 in the many strains examined. However, based on immunoblot, IF, and ELISA of strain 130b, PilA1 is not significantly present in pili, and from mutational analysis, it is also not important for DNA transformation, which agrees with results obtained from analyzing *pilA1* in strain Paris (81). Nonetheless, we hesitate to designate PilA2 as the only major pilin of *L. pneumophila*, since PilA1 might majorly constitute pili under alternative (yet-to-be-defined) growth conditions, including perhaps within biofilms (see discussion below). Clearer from the analyses done by us and Hardy et al. (81) is that PilE is not a major pilin, challenging a viewpoint that had been held for many years (75, 82). Finally, our data found a role for PilT in *L. pneumophila* piliation, which is compatible with the role that PilT has in *L. pneumophila* competence, as shown here and before (81, 86). Overall, the current and recent analyses, which also include the demonstration of FimT/PspA as a DNA-binding protein (86), have revitalized the understanding of what constitutes pili for *L. pneumophila*. However, more refined microscopy and biochemical analyses will be beneficial to elucidate, among other things, the positions of PilE and other minor pilins on the PilA2-T4P, akin to what has been done for T4P of other important pathogens (21, 149).

Without diminishing the value of the structural data summarized above, the most important result of the present study is, arguably, the novel finding that PilA2 promotes *L. pneumophila* growth in low-iron media and iron acquisition/assimilation into the *Legionella* cell. Moreover, given that the phenotype of the *pilA2* mutant was not shared by the other T4P-related mutants examined and that mutants lacking the FeoB Fe^2+^-transporter, LbtC siderophore-transporter, or cytochrome *c* maturation showed greater defects on low-iron media when *pilA2* mutations were introduced into them, the role of PilA2 is likely not due to its (i) incorporation into typical T4P, (ii) maturation from a prepilin form in the periplasm, (iii) engagement with PilS-PilR, or (iv) connection to one of the better known iron-uptake pathways of *L. pneumophila*. This leads to several intriguing hypotheses. In one scenario, the prepilin form of PilA2 within the inner membrane binds or indirectly promotes the binding of periplasmic iron, facilitating a novel form of iron import/assimilation. Compatible with this, AlphaFold-3 modeling suggests that PilA2 might bind ferric iron at an aspartic acid residue in its globular, outward-facing domain (Fig. S7). In another scenario, PilA2 in the inner membrane might engage another signal transduction pathway, which, in turn, activates other iron-acquisition/assimilation genes. Along those lines, minor pilins of *P. aeruginosa* can interact with FimS (in addition to interacting with PilS), which, in turn, signals through AlgR to modulate gene expression (150). As another possibility, some portion of the PilA2 pool might be processed by alternative means and reside in another type of periplasmic, outer membrane, or surface state that engages iron. Finally, it is possible that some percentage of PilA2 is not brought into the inner membrane but remains in the cytoplasm, where it enhances iron assimilation. Since *pilA2* transcript levels and PilA2 protein levels did not appear to increase when *L. pneumophila* was grown in low iron, we further posit that a pre-existing pool of PilA2 is used or repurposed to help the bacterium respond to low-iron conditions. To our knowledge, there are only a few prior studies that draw a connection between T4P and growth in low iron or iron acquisition, and none of them mirror what we have observed. First is the above-mentioned link between PilS/PilR and ferric iron reduction in *G. sulfurreducens* (109, 110). Second are the examples of pilin and other T4P-associated genes having increased expression upon growth in low iron and being repressed by Fur in high iron (151, 152). Third, and in a case more like ours, *Synechocystis* sp. PCC6803 mutants lacking their major pilin have trouble growing in low-iron media and an impaired ability to grow on iron oxides as their sole iron source (153–155). However, in sp. PCC6803 (unlike in *L. pneumophila*), a *pilT* mutant was impaired similarly to the mutant lacking the major pilin, leading to the hypothesis that the cyanobacterial T4P reduce, adsorb, or bind iron (155). Thus, future work aimed at deciphering the mechanism by which PilA2 promotes *L. pneumophila* growth in low iron should advance understanding of pilins, iron acquisition, and perhaps other bacteria that encode type IV pilins.

To our knowledge, there is one prior study examining the role of *L. pneumophila* T4P in biofilms, and in that case, only a *pilE* mutant was examined and found to have an impairment in the early stage of biofilm formation (83). Although a *pilE* mutant did not show an altered phenotype in our biofilm assays, the methodology used in that past study was different from ours in at least three ways, that is, (i) the bacterial inoculum included species of *Flavobacterium, Klebsiella*, and *Pseudomonas* rather than just *L. pneumophila*, (ii) the substratum for the biofilms was steel rather than polystyrene, and (iii) the medium in the chambers was diluted R2A broth rather than BYE broths, that is, nutrient-depleted vs. nutrient-replete. Based on our examination of the *pilA2* mutant vs. WT and complement, PilA2 impedes or limits biofilm formation (and auto-aggregation) by *L. pneumophila*, whereas tests of the *pilA1* mutant vs. WT and complement suggested that PilA1 modestly promotes mono-species biofilms (and auto-aggregation). However, since the *pilT* mutant and *pilQ* mutant showed normal levels of biofilm, the roles of PilA2 and PilA1 cannot be easily explained as effects of T4P. Moreover, because the *pilA2* mutant’s biofilm phenotype was evident when either high-iron or low-iron BYE broth was used as the assay medium, the impact of PilA2 on biofilm formation might also be distinct from the protein’s role on BCYE agar or in CDM broth. Clearly, much more work is needed to determine how PilA2, PilA1, PilE, and other components of T4P are impacting biofilm formation (and auto-aggregation) by *L. pneumophila*. However, it is worth noting that T4P mutants (*pilA2, pilB, pilC,* and *pilN*) of the cyanobacterium *Synechococcus elongatus* exhibit increased biofilm and greater sedimentation (156–159), possibly similar to our *pilA2* mutant’s phenotypes.

In summary, we have confirmed PilA2 as being a major pilin in the competence-associated T4P of *L. pneumophila* and identified a novel role for this type IV pilin in bacterial growth in low iron and iron acquisition/assimilation. That this newfound role of PilA2 appeared to be independent of typical T4P processes and known iron uptake pathways means that further inquiry into PilA2’s locations, interacting partners, and activities should reveal new perspectives regarding pilins and iron acquisition/assimilation. Although our initial investigation of PilS/PilR did not reveal a connection between these proteins and *L. pneumophila* growth on low-iron media, it will be worthwhile for future work to determine what other *Legionella* genes and processes are controlled by the PilS/PilR signal transduction pathway. Finally, it should be noted that the current study began because RNA-seq analysis of a *L. pneumophila cas2* mutant had alerted us to the existence of *pilA2*. Although our subsequent focus was on the roles of PilA2 in piliation, iron acquisition, biofilms, and infection, we did confirm, by qRT-PCR, that *pilA2* (as well as *pilE*) transcripts are increased by the presence of Cas2. We had previously found that *L. pneumophila* Cas2 enhances transcript levels of the small heat shock protein HspC2 and promotes heat tolerance and infection of amoebae (87–89). Thus, future work should ascertain how Cas2 influences *pilA2* expression (in addition to that of *pilE* and *hspC2*) and if it also impacts T4P function, biofilm formation, and growth in low iron. Indeed, work in this arena may help establish a new paradigm for non-canonical activities linked to components of CRISPR-Cas systems (160).

## MATERIALS AND METHODS

### Bacterial strains, media, and extracellular growth

*L. pneumophila* strains 130b (American Type Culture Collection [ATCC] strain BAA-74) and Togus-1 (ATCC 33154) were previously described as were mutants of 130b lacking *proA* (AA200), *pilQ* (NU279), *pilD* (NU272), *lspF* (NU275), *lbtU* (NU383), *lbtC* (NU305), *feoB* (NU269), *ccmC* (NU208), or *cas2* (NU453) (77, 89, 118, 119, 128, 130, 161, 162). These strains and the new mutants made (below) are listed in Table S3A and were routinely grown at 37°C on BCYE agar or in BYE broth (98). Optimal density (OD_660_) readings were used to discern the stages of growth for cultures (136). *E. coli* strain DH5α was used as the host for recombinant plasmids (below) and was routinely grown on LB agar or in LB broth (163).

### Mutant constructions and genetic complementation

Mutants of strain 130b that have deletions in *pilA2* (NU494 and NU495), *pilE* (NU496), *pilA1* (NU497), *pilT* (NU498 and NU499), *pilS* (NU500), or *pilR* (NU501), and a mutant of strain Togus-1 lacking *pilA2* (NU502) were made by allelic exchange utilizing overlap extension PCR (OE-PCR), as before (11, 89, 136, 164). Approximately 1-kb fragments of the 5′ and 3′ regions flanking each of the target ORFs were PCR-amplified from 130b DNA using Platinum SuperFi II DNA polymerase (Thermo Fisher) and primers AB1 and AB2 for 5′ *pilA2*, AB3 and AB4 for 3′ *pilA2*, AB7 and AB8 for 5’*′ pilA1*, AB9 and AB10 for 3′ *pilA1*, AB13 and AB14 for 5*′ pilE*, AB15 and AB16 for 3′ *pilE*, AB19 and AB20 for 5′ *pilT*, AB21 and AB22 for 3′ *pilT*, AB25 and AB26 for 5′ *pilR*, AB27 and AB28 for 3′ *pilR*, AB33 and AB34 for 5′ *pilS*, and AB35 and AB36 for 3′ *pilS*. Sequences for these and all other primers (below) are listed in Table S4. Plasmids are listed in Table S3B. Next, a kanamycin (Kn)-resistance cassette flanked by Flp recombination target sites was PCR-amplified from pKD4 using primers AB5 and AB6 for the eventual mutation of *pilA2*, AB11 and AB12 for *pilA1*, AB17 and AB18 for *pilE*, AB23 and AB24 for *pilT*, AB29 and AB30 for *pilR*, and AB37 and AB38 for *pilS*. OE-PCR was then done to combine the 5′ and 3′ regions of each ORF with the appropriate Kn-resistance cassette. Three micrograms of linear DNA bearing the mutated allele was used for the transformation of WT 130b or Togus-1. Deletion mutants were isolated by plating on BCYE agar containing Kn. Mutants were then verified by performing PCRs using target-specific primers (above) and primers CA43 and CA44, which flank the Kn-resistance cassette. To generate unmarked mutants, the 130b mutants lacking *pilA1*, *pilA2*, *pilE*, or *pilT* were electroporated with pBSFLP, which expresses the FLP recombinase that excises Kn-flanked FRT sites, eliminating Kn-resistance cassettes. Mutants carrying the unmarked deletion and lacking pBSFLP were recovered by plating onto BCYE agar containing 10% sucrose and were screened for loss of Kn and gentamicin (Gm) resistance, as before (89, 165, 166). Mutants were then verified using gene-specific primers (above). To construct a *pilA1 pilA2* mutant of strain 130b (NU503), the *pilA2* 5′ flanking region was amplified with primers AB1 and AB2, the *pilA1* 3′ flanking region with AB9 and AB10, and the Kn-resistance cassette with AB5 and AB12. The mutagenized *pilA1 pilA2* allele was introduced into 130b (as above), and the mutant was verified using primer pair AB1 and AB10. To construct a mutant lacking *lbtC* and *pilA2* (NU504), we introduced the mutagenized *pilA2* allele into the previously made *lbtC* mutant NU383 (130). To construct a *feoB pilA2* mutant (NU505) and a *ccmC pilA2* mutant (NU506), the 5′ and 3′ regions flanking *pilA2* were PCR-amplified using AB1 and AB2 for 5′ *pilA2* and AB3 and AB4 for 3′ *pilA2*. Primers AB5 and AB6 were used to amplify the Gm-resistance cassette from pR6Kgent. After the three fragments were combined by OE-PCR, the mutagenized *pilA2* allele carrying the Gm-resistance cassette was introduced into *feoB* mutant NU269 (118) and *ccmC* mutant NU208 (119). Double mutants were verified using gene-specific primers (above). Complementation of the *pilA2* mutant and *pilA1* mutant was achieved by reintroducing the corresponding genes on a plasmid, as before (11, 136, 167). Briefly, a 411-bp fragment containing the *pilA2* ORF without its native promoter (and no other ORF) was PCR-amplified from 130b genomic DNA using primers AB41 and AB42. Similarly, a 414-bp fragment containing only *pilA1* was PCR-amplified using AB43 and AB44. PCR products were digested with EcoRI and HindIII and cloned into pMMBGent, generating p*pilA2* and p*pilA1*. Plasmids were electroporated into the 130b mutants, and transformants were isolated by plating on BCYE agar containing Gm. Complementation was tested by ectopic expression of *pilA2* and *pilA1* upon induction with 1 mM isopropyl-beta-D-1-thiogalactopyranoside (IPTG).

### Gene transcript analyses

Transcript levels in WT vs. *cas2* mutant *L. pneumophila* were determined by qRT-PCR**,** as before (14, 89). Primers used for these reactions included AB59 and AB60 for the detection of *pilA2* mRNA, AB61 and AB62 for *pilE* transcripts, and JAC35 and JAC36 for *hspC2*. Quantification of transcripts from WT 130b, *pilR* mutant NU501, and pilA2 mutant NU494 grown in media containing different levels of iron was also done as before (14). Briefly, following growth in BYE-Fe broth to mid-log, bacteria were inoculated into CDM broth supplemented with 5 µM ferric pyrophosphate or CDM broth lacking added iron. Upon the cultures growing comparably to mid-log phase, the levels for mRNAs for genes of interest were determined by qRT-PCR. Primer pairs AB59 and AB60 were used for assessing *pilA2*, AL31 and AL32 for *lbtA*, and AL37 and AL38 for *frgA*. Finally, WT 130b and *pilA2* mutant that had plasmid pIroS (168) electroporated into them were grown to mid-log in BYE-Fe broth and then sub-cultured into CDM+Fe broth contained in the wells of a black 96-well microtiter plate (Corning, #CLS3603) and grown at 37°C for 24 h. Bacterial growth was assessed by measuring absorbance at OD_660_, and *frgA* expression was simultaneously monitored by measuring GFP fluorescence. Relative fluorescence units were derived from the ratio of GFP-associated fluorescence levels over the extent of growth (140, 141).

### DNA transformation

*L. pneumophila* competence was determined, as before, with minor modifications (77, 82, 169). Briefly, BYE broth cultures diluted to an OD_660_ of 0.3 were incubated with 5 µg of pGlspGHIJK::Kn (170) at 30°C for 72 h. Dilutions of the transformation mixtures were plated for CFU on BCYE agar containing 25 µg/mL Kn and non-selective BCYE agar. Transformation efficiency was calculated by dividing the CFU/mL obtained on Kn-supplemented BCYE agar by the CFU/mL on standard BCYE agar.

### Immunoblot analysis

To initially track the expression and secretion of PilA2 and PilA1, a FLAG tag was added to the C-terminus of each protein, as we did before for other proteins (11). Plasmids encoding FLAG-tagged PilA2 or PilA1 were made by PCR-amplifying the respective genes from 130b DNA using primers AB41 and AB45 for *pilA2* and AB43 and AB46 for *pilA1*. PCR products were digested with EcoRI and HindIII and cloned into Gm-resistant pMMBGent. The new plasmids p*pilA2*-FLAG and p*pilA1*-FLAG were confirmed using vector-specific primers OR77 and OR78 and sequencing. Each plasmid was electroporated into WT 130b and *proA* mutant AA200, and transformants were isolated on BYCE agar containing Gm. Strains containing plasmids were grown in BYE broth with 1 mM IPTG (to induce the expression of the tagged proteins) to the early stationary phase. Ten milliliters of the culture were vortexed at max speed for 1 min. Pellets were harvested by centrifuging at 4,750 × *g* for 20 min, and the supernatants containing sheared-off pili were filter-sterilized. As before (11, 14, 171), the supernatants were concentrated (33-fold) by isopropanol precipitation. Following suspension in Laemmli buffer, supernatant and pellet samples were subjected to SDS-PAGE and immunoblotting as before (11). Membranes were first incubated with 1% bovine serum albumin (BSA) in Tris-buffered saline containing Tween-20 (TBST). Later, membranes were incubated with a 1:5,000 dilution of rabbit anti-FLAG antibody (Invitrogen) in BSA-TBST and then a 1:10,000 dilution of IRDye 680 goat anti-rabbit IgG antibody (LI-COR Biosciences) in BSA-TBST. Finally, to assess the effect of iron on expression of PilA2 from its chromosomal locus, 130b encoding PilA2-FLAG (NU507, below) was grown in either CDM broth supplemented with 5 µM ferric pyrophosphate or CDM broth lacking added iron. Cell lysates were subjected to SDS-PAGE, with equal amounts of protein loaded per sample, and immunoblotted utilizing, as primary antibody, either the anti-FLAG antibody (above) or rabbit anti-ICDH antibodies (128) at 1:1,000 dilution. Densitometry was performed in ImageJ, in which the intensities of the bands of interest (e.g., PilA2-FLAG) were quantified by measuring integrated density using identical rectangular regions of interest for each band, followed by background subtraction (172–174). To further normalize across samples, the PilA2-FLAG intensity for each lane was divided by the corresponding ICDH band intensity from the same lane.

### IF microscopy

To visualize T4P on the surface of *L. pneumophila* strain 130b, we performed IF microscopy, as before (81), albeit with some modifications. This process entailed examining WT and *proA* mutant carrying p*pilA2*-FLAG or p*pilA1*-FLAG (above) and WT and a *pilT* mutant expressing FLAG-tagged PilA2 or FLAG-tagged PilA1 from their native chromosomal locus. In the latter case, *pilA2*-FLAG and *pilA1*-FLAG were PCR-amplified from p*pilA2*-FLAG and p*pilA1*-FLAG with primers AB51 and AB52 for *pilA2*-FLAG and AB57 and AB58 for *pilA1*-FLAG. Then, ~1 kb fragments of the 5′ and 3′ regions flanking *pilA2* and *pilA1* were PCR amplified from 130b genomic DNA using primers AB47 and AB48 for 5′ *pilA2*, AB49 and AB50 for 3′ *pilA2*, AB53 and AB54 for 5′ *pilA1*, and AB55 and AB56 for 3′ *pilA1*. OE-PCR was performed to combine the 5′ and 3′ regions of each ORF with the ORF containing the FLAG tag. PCR products were then digested with NotI and SalI and cloned into pSR47S Kn (175, 176), generating p*pilA2*-FLAG-int and p*pilA1*-FLAG-int. Plasmid DNA was transformed into the 130b strains, and transformants were isolated on BCYE agar supplemented with Kn. Resulting colonies were plated on BCYE agar containing 10% sucrose to select for loss of plasmid. The strains containing chromosomal FLAG-tags (NU507 for *pilA2*-FLAG and NU508 for *pilA1*-FLAG) were verified by sequencing using primers AB47 and AB50 for *pilA2*-FLAG and AB53 and AB56 for *pilA1*-FLAG. *pilT* was deleted (as above) from these strains to further test for the effect of PilT on piliation, resulting in strain NU509 for *pilA2*-FLAG and NU510 for *pilA1*-FLAG. To obtain bacterial samples for IF microscopy, the strains ectopically expressing the FLAG-tagged pilins were grown on BCYE agar containing 1 mM IPTG for 2 days at 37°, whereas strains having chromosomally encoded FLAG-tagged pilin were grown on BCYE agar for 5 days at 37°C. Bacteria were suspended in PBS using a cotton tip applicator and gently mixed. After fixation with 4% paraformaldehyde (PFA) (ThermoFisher), the cells were added to coverslips that had been treated with 0.01% poly-*L*-lysine (EMD Millipore). After 30 min of incubation, the coverslips were washed with PBS and then incubated with PBS containing 1% BSA (BSA-PBS). The coverslips were then treated with a 1:5,000 dilution of rabbit anti-FLAG antibody (above) in BSA-PBS, washed twice, and then incubated with a 1:20,000 dilution of goat anti-rabbit Alexa Fluor 488-conjugated secondary antibody (Invitrogen #A27034) in BSA-PBS. After a final set of washes, the coverslips were mounted and imaged with an epifluorescence microscope (Nikon Eclipse 90i) using the FITC channel (excitation, 495 nm; emission 520 nm). At least 1,000 bacterial cells were visualized per strain per experiment.

### Whole-cell ELISA

Immunodetection of pilin proteins on the surface of *L. pneumophila* was done analogously to the analyses of other pilins and surface proteins, with some modifications (177–179). Colonies of 130b WT and *pilT* mutant encoding FLAG-tagged PilA2 or PilA1 from their chromosomal locus (above) that had grown on BCYE agar for 5 days at 37°C were suspended in PBS to an OD_660_ of 0.3, and 200 µL of aliquots was transferred into the wells of a 96-well flat-bottom polystyrene microtiter plate. After the plates were centrifuged at 2,500 × *g* for 13 min, supernatants were removed, and the plate was air-dried. Next, bacteria were fixed in 4% PFA, washed three times with PBS, and then 200 µL of blocking buffer (PBS + 1% BSA) was added. This was followed by a 1 h incubation with 100 µL of rabbit anti-FLAG antibodies (above) diluted 1:1,000 in blocking buffer. After washing, 100 µL of goat anti-rabbit conjugated horseradish peroxidase antibody (Cell Signaling, #7074S) diluted 1:1,000 in blocking buffer was added for 1 h. After washing, 3,3′,5,5′-tetramethylbenzidine (Invitrogen) was added for 15 min. Reactions were stopped with 2 N sulfuric acid, and absorbances were measured at 450 nm.

### Assessments of bacterial growth in media containing differing amounts of iron

To assess colony size, strains were grown for 3–5 days at 37°C on BCYE agar containing differing amounts of iron. Photos were taken using a Nikon SMZ-10A stereomicroscope, and the diameters of the colonies were measured using ImageJ. To determine CFU numbers within colonies, colonies were suspended in PBS, vortexed at the maximum setting for 1 min, diluted in PBS, and plated for CFU on standard BCYE agar. Strains were also assessed for their eop on BCYE agar with differing amounts of available iron, as before (14, 128, 130, 136). To further measure growth under iron-depleted conditions, strains were first grown in CDM-Fe broth to mid-log phase, at which point there were no growth differences. After being diluted into fresh CDM-Fe broth with or without added metals to an OD_660_ of 0.2, growth was then measured in microtiter plates using a SpectraMax iD3 plate reader (Molecular Devices). Alternatively, bacteria were first grown in BYE broth with or without added iron to mid-log phase, diluted into fresh BYE broth with or without added iron to an OD_660_ of 0.2, and growth was assessed as above.

### Streptonigrin-sensitivity assay

After *L. pneumophila* strains were grown in CDM-Fe broth to mid-log phase, bacteria were diluted to an OD_660_ = 0.3 in CDM-Fe broth. One milliliter of aliquot was mixed with 5 µL of DMSO containing (fresh) streptonigrin, resulting in a final streptonigrin concentration of 5 µM (Sigma-Aldrich, #S1014). Samples were incubated at 37°C with shaking. At *t* = 0 and 1 h, aliquots taken from the tubes were diluted in PBS and plated for CFU on BCYE agar, and then, the percent bacterial survival was calculated. Control experiments determined that DMSO alone did not alter bacterial survival in the 1-h incubation period.

### Intracellular infection, biofilm, and auto-aggregation assays

*L. pneumophila* growth in *A. castellanii* (ATCC 30234) was done as before (118, 136, 180). Briefly, monolayers of *A. castellanii* were infected with legionellae at an MOI = 0.1, and then immediately (i.e., *t* = 0) and at 24, 48, and 72 h post-inoculation, aliquots taken from the culture supernatants were assessed for bacterial numbers by plating for CFU on BCYE agar. Because *L. pneumophila* does not replicate in the assay medium, increases in CFU are due to bacterial growth in the amoebae. For infection with iron depletion, 25 µM DIP (Acros Organics) was added 24 h prior to inoculation and every 24 h thereafter. *L. pneumophila* biofilm formation on plastic microtiter plates, as measured by crystal violet staining, was done as before (11, 14, 136). Briefly, following 3 days of growth on BCYE agar at 37°C, *L. pneumophila* strains were resuspended to an OD_660_ of ~0.2 in either BYE broth containing its standard amount of added iron (BYE + Fe) or BYE broth lacking the iron supplement (BYE – Fe), and then, the suspensions were added into the wells of a 96-well, polystyrene microtiter plate. After 1 day at 37°C or 2 days at 30°C, biofilm formation was assessed by staining, with absorbance measured at 600 nm. *L. pneumophila* auto-aggregation was assessed as before (11, 14). Briefly, following growth on BCYE agar at 30°C, strains were suspended in 10% BYE broth (in glass tubes) to an OD_660_ of ~1.0, and bacterial sedimentation at 30°C was assessed by measuring drops in the OD_660_ of the statically incubated suspensions.

### *In silico* analyses

BLASTP at the NCBI was used to detect homologs of PilA2 and PilA1 in *L. pneumophila* strains, other *Legionella* species, and the general database, as well as to determine the percentage identity between PilA1 and PilA2. PilA2/PilA1-related proteins encoded by DIG species were organized on a phylogenetic tree, as before (11, 14). Predicted structures for PilA2, PilA1, and PilE were made using AlphaFold 3 (181) and rendered with ChimeraX (182). To discern relationships to known structures, these structures were submitted to the DALI server (183), as before (11, 14, 89, 163).

### Statistical methods

Experiments used more than three technical replicates. Numerical data were demonstrated as means and standard deviations. Unless stated otherwise, *P* values were obtained using Student’s *t*-tests. Repeat experiments, that is, biological replicates, were done, as noted in the figure legends.

## SUPPLEMENTAL MATERIAL

Supplemental Material mbio.00596-26-s0001.pdf Tables S1-S4 and Fig. S1-S7.

ASM does not own the copyrights to Supplemental Material that may be linked to, or accessed through, an article. The authors have granted ASM a non-exclusive, world-wide license to publish the Supplemental Material files. Please contact the corresponding author directly for reuse.
