# Supplementary material for: Major type IV pilin of Legionella pneumophila enhances bacterial iron acquisition/assimilation, independently of its role in piliation
Source: mBio. 2026 Apr 24;17(5):e00596-26. doi: 10.1128/mbio.00596-26 (PMC13170364; doi:10.1128/mbio.00596-26)
Supplement: Supplemental Material — Tables S1-S4 and Fig. S1-S7. [file mbio.00596-26-s0001.pdf]

TABLE S1. BLASTP results indicating the presence of *pilA2* and *pilA1* within sequenced strains of *L. pneumophila*

			% identity relative to the proteins of 130b	
**Accession Number**	**Strain**	**Source**	**PilA2**	**PilA1**
GCF_041734345.1	130b	clinical	100	100
GCF_000048645.1	Paris	clinical	97.79	97.81
GCF_000048665.1	Lens	clinical	89.05	89.78
GCF_000008485.1	Philadelphia-1	clinical	97.06	97.08
GCF_002002645.1	Sudbury	clinical	99.26	97.81
GCF_000404245.1	Thunder Bay	clinical	97.06	97.08
GCF_001886795.1	Detroit-1	clinical	100	100
GCF_000239175.1	ATCC 43290	clinical	97.06	97.08
GCF_000347615.1	LPE509	clinical	97.06	97.08
GCF_000306865.1	Lorraine	clinical	97.79	97.81
GCF_002002625.1	Mississauga	clinical	99.26	97.81
GCF_001592705.1	Toronto-2005	clinical	89.05	86.86
GCF_000092545.1	Corby	clinical	84.4	86.86
GCF_000586295.1	ATTC 33215	clinical	97.06	97.08
GCF_000092625.1	Alcoy	clinical	99.26	97.81
GCF_000465675.1	Leg01/11	clinical	99.26	97.81
GCA_015989285.1	AZ00029759	clinical	99.26	97.81
GCF_001753085.1	C9_S	clinical	97.06	97.08
GCF_001766275.1	FFI102	clinical	89.05	89.78
GCF_004170265.1	NMB001870	clinical	89.05	89.78
GCF_004169905.1	NMB001868	clinical	89.05	89.78
GCA_015986385.1	A19030476	clinical	97.79	97.81
GCA_015894685.1	D1169	clinical	99.26	97.81
GCA_015893395.1	D5035	clinical	97.06	97.98
GCF_002813735.1	D5945	clinical	100	100
GCF_002813715.1	D6026	clinical	100	100
GCF_900452685.1	NCTC12000	clinical	99.26	97.81
GCF_001549925.1	PtVF66/2014	clinical	100	100
GCA_015894625.1	D1405	clinical	99.26	97.81
GCA_015895145.1	D3294	clinical	99.26	97.81
GCA_015895365.1	D4700	clinical	99.26	97.81
GCA_015950945.1	F4455	environmental	97.06	96.35
GCA_001766295.1	FFI103	environmental	89.05	89.78
GCA_001766355.1	FFI329	environmental	89.05	89.78
GCA_015975845.1	Isolate 4368	environmental	99.26	97.81

GCA_015971365.1	Isolate 4788	environmental	97.06	96.35
GCA_015970645.1	Isolate 4825	environmental	97.06	96.35
GCA_015979325.1	LG51	environmental	97.06	96.35
GCA_000347615.1	LPE509	environmental	97.06	97.08
GCA_001583655.1	SH003	environmental	97.06	96.35
GCA_015943225.1	CL20-200363	environmental	99.26	97.81
GCA_001886835.1	Dallas-1E	environmental	100	100
GCA_003205115.1	GC03	environmental	99.26	97.81
GCA_003205045.1	GC04	environmental	99.26	97.81
GCA_003205035.1	GC05	environmental	99.26	97.81
GCA_015977605.1	Isolate 3969	environmental	97.06	96.35
GCA_015976285.1	Isolate 4343	environmental	97.06	96.35
GCA_015970705.1	Isolate 4809	environmental	97.06	96.35
GCA_015971125.1	Isolate 4826	environmental	97.06	96.35
GCA_900637585.1	NCTC12273	environmental	97.79	95.62
GCA_001677115.2	Pontiac	environmental	89.05	86.86
GCA_015946125.1	CL20-200126	environmental	99.26	97.81
GCA_016021295.1	CL20-200318	environmental	97.79	97.08
GCA_015943185.1	CL20-200376	environmental	99.26	97.81
GCA_017716275.1	SU18-MZ1	environmental	96.32	92.70
GCA_017676575.1	SU18-MZ2	environmental	96.32	92.70

TABLE S2. BLASTP results indicating the relatedness of PilA2 to proteins outside of the deep-branching intracellular γ-proteobacteria

A. Proteins (i.e., hypothetical pilins) most related to PilA2 of *L. pneumophila*

Species *	Coverage	E value	Identity	Accession
Ideonella lacteola	98%	3.00E-49	60.14%	WP_341425320.1
Pseudoalteromonas atlantica	99%	5.00E-49	61.54%	WP_318299767.1
Spartinivicinus ruber	100%	6.00E-48	61.74%	WP_329604954.1
Ectothiorhodospira marina	99%	4.00E-47	57.97%	WP_090255633.1
Variovorax gossypii	98%	8.00E-46	54.04%	WP_126473919.1
Nitrosomonas mobilis	99%	3.00E-45	57.53%	WP_090286186.1
Quisquiliibacterium transsilvanicum	98%	1.00E-44	60.43%	WP_183970459.1
Roseateles rivi	98%	7.00E-44	56.83%	WP_394458879.1
Modicisalibacter tunisiensis	98%	2.00E-42	55.86%	WP_318010290.1
Aquabacterium olei	98%	1.00E-41	56.76%	WP_109034435.1
Pseudomonas viridiflava	98%	3.00E-41	58.27%	WP_025993964.1
Microbulbifer hainanensis	99%	3.00E-41	54.00%	WP_193161308.1
Spongiibacter nanhainus	98%	9.00E-41	56.43%	WP_198570571.1
Acinetobacter junii	98%	1.00E-40	53.66%	WP_212638999.1
Arsukibacterium perlucidum	99%	3.00E-40	53.42%	WP_019675192.1
Thiobacter aerophilum	98%	4.00E-40	65.03%	WP_347307121.1
Allochromatium humboldtianum	98%	5.00E-40	55.86%	WP_176976014.1
Rehaibacterium terrae	98%	5.00E-40	55.15%	WP_281379538.1
Alishewanella jeotgali	99%	5.00E-40	55.32%	WP_008950231.1
Sansalvadorimonas verongulae	98%	2.00E-39	55.56%	WP_155163297.1
Cupriavidus basilensis	99%	2.00E-39	56.95%	WP_277820352.1
Simplicispira psychrophila	99%	4.00E-39	56.55%	WP_281174765.1
Zooshikella ganghwensis	98%	8.00E-39	55.56%	WP_212715763.1
Piscinibacter gummiphilus	98%	1.00E-38	52.94%	WP_085748872.1
Silvimonas terrae	98%	1.00E-38	55.41%	WP_184102033.1
Methylohalomonas lacus	98%	2.00E-38	52.52%	WP_259055856.1
Polaromonas glacialis	99%	2.00E-38	51.25%	WP_269635728.1
Aromatoleum diolicum	98%	3.00E-38	52.35%	WP_169258949.1
Litorivicinus lipolyticus	98%	3.00E-38	54.42%	WP_153714430.1
Arenimonas maotaiensis	98%	6.00E-38	51.80%	WP_188448437.1
Thiopseudomonas acetoxidans	99%	8.00E-38	52.52%	WP_289411672.1
Desulfotalea psychrophila	99%	2.00E-37	56.20%	WP_011188693.1
Ralstonia edaphi	98%	3.00E-37	51.25%	WP_316900089.1
Lysobacter koreensis	99%	4.00E-37	60.71%	WP_386812149.1
Melaminivora alkalimesophila	98%	7.00E-37	57.25%	WP_277422519.1

Amantichitinum ursilacus	98%	1.00E-35	47.27%	WP_053938512.1
Marinagarivorans cellulosilyticus	98%	2.00E-35	52.78%	WP_236985603.1
Hydrogenophaga palleronii	98%	3.00E-35	53.16%	WP_066271701.1
Pandoraea thiooxydans	98%	4.00E-35	49.36%	WP_047214930.1
Alloalcanivorax profundimaris	98%	1.00E-34	53.79%	WP_194296278.1
Comamonas sediminis	98%	1.00E-34	47.88%	WP_369460188.1
Noviherbaspirillum aerium	98%	4.00E-34	57.66%	WP_318841027.1
Oceanococcus atlanticus	98%	7.00E-34	51.45%	WP_083560126.1
Dechloromonas agitata	98%	9.00E-34	57.14%	WP_027457762.1
Thiothrix lacustris	99%	1.00E-33	58.09%	WP_028490495.1
Ventosimonas gracilis	99%	1.00E-33	51.39%	WP_275574870.1
Corallincola platygyrae	98%	1.00E-33	54.23%	WP_345339653.1
Gilvimarinus chinensis	99%	3.00E-33	46.50%	WP_020207947.1
Novilysobacter spongiicola	99%	4.00E-33	58.87%	WP_078758386.1
Psychrobacter pacificensis	98%	5.00E-33	45.73%	WP_212563960.1
Neisseria cinerea	98%	5.00E-33	56.43%	WP_108044216.1
Stagnimonas aquatica	99%	6.00E-33	59.29%	WP_123210247.1
Spectribacter acetivorans	99%	6.00E-33	57.66%	WP_349255456.1
Xanthomonas pisi	99%	6.00E-33	48.53%	WP_104612286.1
Chromobacterium sinusclupearum	98%	8.00E-33	46.39%	WP_103317638.1
Uruburuella testudinis	98%	9.00E-33	50.71%	WP_244785096.1
Ottowia thiooxydans	98%	1.00E-32	52.48%	WP_028604253.1
Marichromatium gracile	98%	1.00E-32	55.56%	WP_132230655.1
Sphaerotilus sulfidivorans	98%	1.00E-32	46.20%	WP_149505346.1
Marinicella gelatinilytica	99%	1.00E-32	50.37%	WP_267182972.1
Methylomicrobium agile	99%	1.00E-32	51.47%	WP_031431153.1
Shewanella marina	98%	1.00E-32	51.45%	WP_304621829.1
Pseudorhodoferax aquiterrae	98%	2.00E-32	50.35%	WP_308433234.1
Aquitalea palustris	98%	2.00E-32	54.93%	WP_103524441.1
Niveibacterium umoris	98%	2.00E-32	48.77%	WP_183634493.1
Marinobacter qingdaonensis	98%	3.00E-32	51.75%	WP_322855871.1
Luteimonas vadosa	98%	4.00E-32	53.96%	WP_345293498.1
Silanimonas lenta	98%	9.00E-32	56.34%	WP_028770315.1
Trinickia violacea	99%	5.00E-32	44.00%	WP_137331189.1
Extensimonas vulgaris	99%	8.00E-32	50.36%	WP_114482850.1
Morococcus cerebrosus	99%	1.00E-31	48.99%	WP_039409671.1
Simiduia litorea	98%	1.00E-31	47.31%	WP_390593921.1
Stenotrophomonas oahuensis	99%	1.00E-31	55.32%	WP_311192387.1
Saccharophagus degradans	98%	2.00E-31	56.62%	WP_011467347.1
Hahella ganghwensis	99%	2.00E-31	48.23%	WP_020409620.1

Cognatiluteimonas lumbrici	98%	2.00E-31	47.22%	WP_342776931.1
Methylibium petroleiphilum	98%	2.00E-31	47.31%	WP_083767920.1
Aromatoleum petrolei	98%	4.00E-31	48.15%	WP_169206171.1
Malikia spinosa	98%	4.00E-31	54.93%	WP_105729274.1
Acidovorax radicis	98%	6.00E-31	45.73%	WP_010464532.1
Pseudomaricurvus hydrocarbonicus	98%	6.00E-31	52.86%	WP_167183231.1
Pseudoxanthomonas mexicana	99%	6.00E-31	54.29%	WP_187573329.1
Nitrosococcus watsonii	98%	7.00E-31	48.55%	WP_013221397.1
Collimonas arenae	98%	9.00E-31	47.55%	WP_082797997.1
Piscinibacter gummiphilus	99%	2.00E-30	47.52%	WP_085748873.1
Pseudogulbenkiania subflava	98%	2.00E-30	48.28%	WP_085277761.1
Herbaspirillum hiltneri	98%	2.00E-30	48.00%	WP_053195944.1
Burkholderia stabilis	99%	2.00E-30	42.86%	WP_122168587.1
Alcanivorax hongdengensis	99%	2.00E-30	45.45%	WP_040296993.1
Andreprevotia lacus	79%	3.00E-30	57.41%	WP_084093001.1
Rhodoferax koreensis	99%	3.00E-30	47.52%	WP_076203897.1
Paraburkholderia megapolitana	90%	3.00E-30	48.95%	WP_091011122.1
Sphaerotilus uruguayifluvii	98%	4.00E-30	50.36%	WP_173806020.1
Nevskia ramosa	98%	6.00E-30	48.55%	WP_028474941.1
Aliikangiella marina	98%	6.00E-30	54.55%	WP_142943781.1
Rivibacter subsaxonicus	98%	6.00E-30	46.39%	WP_130431413.1
Rubrivivax albus	98%	7.00E-30	52.24%	WP_128198027.
Neptunomonas marina	99%	9.00E-30	59.86%	WP_127692256.1
Silvimonas soli	98%	1.00E-29	46.86%	WP_283151119.1
Parathalassolituus penaei	95%	1.00E-29	51.03%	WP_283172503.1
Ramlibacter paludis	98%	1.00E-29	47.45%	WP_275699411.1
Thauera butanivorans	98%	1.00E-29	45.78%	WP_418648401.1
Variovorax dokdonensis	99%	2.00E-29	51.08%	WP_286659739.1
Kistimonas asteriae	99%	2.00E-29	47.83%	WP_211827863.1
Xenophilus arseniciresistens	98%	2.00E-29	49.31%	WP_271430097.1
Methylosarcina fibrata	99%	2.00E-29	46.67%	WP_020561648.1
Alloalcanivorax venustensis	98%	3.00E-29	50.34%	WP_414434047.1
Halomonas salifodinae	98%	3.00E-29	47.22%	WP_346060576.1
Massilia glaciei	98%	7.00E-29	52.55%	WP_370660178.1
Zeimonas arvi	98%	7.00E-29	53.28%	WP_222707857.1
Ramlibacter albus	98%	8.00E-29	51.85%	WP_187080084.1
Tepidiphilus succinatimandens	98%	9.00E-29	50.36%	WP_141056401.1
Herbaspirillum camelliae	98%	1.00E-28	45.68%	WP_075256889.1
Caballeronia arvi	96%	1.00E-28	46.36%	WP_096032026.1

* For brevity, only a single representative species per genus is listed. The list is also not exhaustive because it does not include the results that have *E* values between 1e-28 and borderline significance.

B. Relationships between *L. pneumophila* PilA2 and some of the known major type IV pilins

**Bacteria**	**Coverage**	**E value**	**Identity**	**Accession**
Burkholderia pseudomallei	90%	5.00E-30	46.85%	WP_076953316.1
Pseudomonas aeruginosa	98%	9.00E-27	42.37%	NP_253215.1
Neisseria gonorrhoeae	98%	2.00E-27	36.54%	P02974.2
Neisseria meningitidis	87%	5.00E-26	43.20%	P57039.1
Myxococcus xanthus	95%	6.00E-20	34.21%	3JC8_A1
Vibrio cholerae	98%	1.00E-18	36.03%	WP_057563244.1

TABLE S3. *L. pneumophila* strains and plasmids used in this study

A. *L. pneumophila* strains

**Strain**	**Description**
130b	Clinical isolate, serogroup 1
AA200	*proA* mutant of 130b
NU208	*ccmC* mutant of 130b
NU269	*feoB* mutant of 130b
NU272	*pilD* mutant of 130b
NU275	*lspF* mutant of 130b
NU279	*pilQ* mutant of 130b
NU305	*lbtC* mutant of 130b
NU383	*lbtU* mutant of 130b
NU453	*cas2* mutant of 130b
NU494	*pilA2* mutant of 130b
NU495	*pilA2* mutant of 130b
NU496	*pilE* mutant of 130b
NU497	*pilA1* mutant of 130b
NU498	*pilT* mutant of 130b
NU499	*pilT* mutant of 130b
NU500	*pilS* mutant of 130b
NU501	*pilR* mutant of 130b
NU503	*pilA1 pilA2* mutant of 130b
NU504	*lbtC pilA2* mutant of 130b
NU505	*feoB pilA2* mutant of 130b
NU506	*ccmC pilA2* mutant of 130b
NU507	130b encoding chromosomal *pilA2*-FLAG
NU508	130b encoding chromosomal *pilA1*-FLAG
NU509	*pilT* mutant encoding chromosomal *pilA2*-FLAG
NU510	*pilT* mutant encoding chromosomal *pilA1*-FLAG
NU294 (p*pilA2*)	Complemented *pilA2* mutant
NU297 (p*pilA1*)	Complemented *pilA1* mutant
130b (p*pilA2*-FLAG)	130b harboring p*pilA2*-FLAG
AA200 (p*pilA2*-FLAG)	AA200 harboring p*pilA2*-FLAG
130b (p*pilA1*-FLAG)	130b harboring p*pilA1*-FLAG
AA200 (p*pilA1*-FLAG)	AA200 harboring p*pilA1*-FLAG
130b (pIroS)	130b harboring pIroS
NU294 (pIroS)	NU294 harboring pIroS
Togus-1	Clinical isolate, serogroup 2
NU502	*pilA2* mutant of Togus-1

B. Plasmids

**Plasmid**	**Description**
pMMBGent	Cloning plasmid carrying a gentamicin-resistance cassette
p*pilA2*	*pilA2* cloned into pMMBGent
p*pilA1*	*pilA1* cloned into pMMBGent
p*pilA2*-FLAG	*pilA2*-FLAG cloned into pMMBGent
p*pilA1*-FLAG	*pilA1*-FLAG cloned into pMMBGent
pSR47S	Suicide vector carrying a kanamycin-resistance cassette
p*pilA2*-FLAG-int	*pilA2*-FLAG cloned into pSR47S
p*pilA1*-FLAG-int	*pilA1*-FLAG cloned into pSR47S
pIroS	Plasmid encoding GFP under the control of the *frgA* promoter
pGlspGHIJK::Kn	Kanamycin-resistance cassette-inactivated *lspGHIJK* locus cloned into pGEM-T Easy

TABLE S4. Primers used in this study

Name	Description	Sequence (5' to 3') *
AB1	pilA2 5'F	GAGCAGCACTAAAGCAAGAACG
AB2	pilA2 5'R	GAAGCAGCTCCAGCCTACACACGATAATCGCTACGACTATCATC
AB3	pilA2 3'F	TAAGGAGGATATTCATATGCAAATACCGACCTGCCAGTTGC
AB4	pilA2 3'R	GGACGCATGCAAACGTTACTTG
AB5	pilA2_KanF	GATGATAGTCGTAGCGATTATCGTGTGTAGGCTGGAGCTGCTTC
AB6	pilA2_KanR	GCAACTGGCAGGTCGGTATTTGCATATGAATATCCTCCTTA
AB7	pilA1 5'F	CTGGTAGTTGGCATTATGCTTGC
AB8	pilA1 5'R	GAAGCAGCTCCAGCCTACACAATAGCGGCAACGATCATCAGC
AB9	pilA1 3'F	TAAGGAGGATATTCATATGGATTGCACTGGTGGTACCCTGATTAG
AB10	pilA1 3'R	CTTGAGGTGATGGGAAGGCATG
AB11	pilA1_KanF	GCTGATGATCGTTGCCGCTATTGTGTAGGCTGGAGCTGCTTC
AB12	pilA1_KanR	CTAATCAGGGTACCACCAGTGCAATCCATATGAATATCCTCCTTA
AB13	pilE 5'F	TGA**GCGGCCGC**GATTGCAAGCCGATCCTGTTAC (NotI)
AB14	pilE 5'R	GAAGCAGCTCCAGCCTACACACCGATTGTTTCATACGGCTATTC
AB15	pilE 3'F	TAAGGAGGATATTCATATGGCAACCAGAATGCTGGAATCC
AB16	pilE 3'R	GCA**GTCGAC**CACCGATACTCGCTATGGTATTGG (SalI)
AB17	pilE_KanF	GAATAGCCGTATGAAACAATCGGTGTGTAGGCTGGAGCTGCTTC
AB18	pilE_KanR	GGATTCCAGCATTCTGGTTGCCATATGAATATCCTCCTTA
AB19	pilT 5'F	TGA**GCGGCCGC**CGAACTATGGTCGAACCTGC (NotI)
AB20	pilT 5'R	GAAGCAGCTCCAGCCTACACAGCCAATAATTCCGCGATATCCA
AB21	pilT 3'F	TAAGGAGGATATTCATATGCACACTGCTCATGAGGCTG
AB22	pilT 3'R	GCA**GTCGAC**CAACTCATAGCCGTACCGAAAG (SalI)
AB23	pilT_KanF	TGGATATCGCGGAATTATTGGCTGTGTAGGCTGGAGCTGCTTC
AB24	pilT_KanR	CAGCCTCATGAGCAGTGTGCATATGAATATCCTCCTTA
AB25	pilR 5'F	GTAGCTATGAGTTGGCAGGAATG
AB26	pilR 5'R	GAAGCAGCTCCAGCCTACACACTGGTTCATCATCTATAACAAGCAC
AB27	pilR 3'F	TAAGGAGGATATTCATATGGCTTTCGAACTCTCCGTTATCG
AB28	pilR 3'R	GAGCGCTTCTGGATTGAATACG
AB29	pilR_KanF	GTGCTTGTTATAGATGATGAACCAGTGTGTAGGCTGGAGCTGCTTC
AB30	pilR_KanR	CGATAACGGAGAGTTCGAAAGCCATATGAATATCCTCCTTA
AB31	pilR_seq_F	GAACAATTGTGAGCGAATGAACAGG
AB32	pilR_seq_R	CCAGAGGTAGACCAAAGACTTTG
AB33	pilS 5'F	ATTGGTATGAAAAAGCAGCG
AB34	pilS 5'R	GAAGCAGCTCCAGCCTACACACCTTTTCTAATCCATTAGATTATTC
AB35	pilS 3'F	TAAGGAGGATATTCATATGCCATCGGATGAACTTTTAAT
AB36	pilS 3'R	ATCCACAGGAACTTCATATA
AB37	pilS_KanF	GAATAATCTAATGGATTAGAAAAGGTGTGTAGGCTGGAGCTGCTTC
AB38	pilR_KanR	ATTAAAAGTTCATCCGATGGCATATGAATATCCTCCTTA
AB39	pilS_Seq_F	AAAAGCAGCTATTAGTTGGT

AB40	pilS_seq_R	TTCCATTAAAGTTGGGACAC
CA43	Kan Internal F	GGGCACAACAGACAATCGGC
CA44	Kan Internal R	CTCTTCAGCAATATCACGGGTAGCC
AB41	pilA2_pmmBGent_F	GCATGA**GAATTC**ATGAGACAAAAGGGTTTTACTTTAATTGAATTGATG (EcoRI)
AB42	pilA2_pmmBGent_R	CGTACT**AAGCTT**TTATGGTCTGCAACTGGCAGGTCGGTATTTGG (HindIII)
AB43	pilA1_pmmBGent_F	GCATGA**GAATTC**ATGAGAACGAAAGGATTTACTTTAATCGAGCTGATG (EcoRI)
AB44	pilA1_pmmBGent_R	CGTACT**AAGCTT**TTAAGGGCGGCAGTAGGCTGGACGATATTTACTAATC (HindIII)
AB45	pilA2_pmmBGent_Flag_R	CGTACT**AAGCTT**TTACTTGTCGTCATCGTCTTTGTAGTCTGGTCTGCAACTGGCAGGTCGGTATTTGG (HindIII)
AB46	pilA1_pmmBGent_Flag_R	CGTACT**AAGCTT**TTACTTGTCGTCATCGTCTTTGTAGTCAGGGCGGCAGTAGGCTGGACGATATTTACTAATC (HindIII)
OR77	Vector Fwd	TCGGCTCGTATAATGTGTGG
OR78	Vector Rev	ACCGCTTCTGCGTTCTGATT
AB47	pilA2_cFlag_ 5'F	TGA**GCGGCCGC**GAGGACAGTTCAGCAGCGACTAAAG NotI)
AB48	pilA2_cFlag_ 5'R	CAATTAAAGTAAAACCCTTTTGTCTCATGACCATCTCCATAGTTAACTATCCATTAG
AB49	pilA2_cFlag_ 3'F	GACTACAAAGACGATGACGACAAGTAATCGGATAAAGCAACTATAAAAACCGC
AB50	pilA2_cFlag_ 3'R	GCA**GTCGAC**CCTCTAGCTGAGGTAGCTAAC (SalI)
AB51	pilA2_cFlag fwd	CTAATGGATAGTTAACTATGGAGATGGTCATGAGACAAAAGGGTTTTACTTTAATTG
AB52	pilA2_cFlag rev	GCGGTTTTTATAGTTGCTTTATCCGATTACTTGTCGTCATCGTCTTTGTAGTC
AB53	pilA1_cFlag_ 5'F	TGA**GCGGCCGC**CTGGTAGTTGGCATTATGCTTGC (NotI)
AB54	pilA1_cFlag_ 5'R	CGATTAAAGTAAATCCTTTCGTTCTCATAACGTCACCTGGCATTGCATCTAAC
AB55	pilA1_cFlag_ 3'F	GACTACAAAGACGATGACGACAAGTAATCTTGATTGATTTTTTTAGCCACTAAC
AB56	pilA1_cFlag_ 3'R	GCA**GTCGAC**CTTGAGGTGATGGGAAGGCATG (SalI)
AB57	pilA1_cFlag fwd	GTTAGATGCAATGCCAGGTGACGTTATGAGAACGAAAGGATTTACTTTAATCG
AB58	pilA1_cFlag rev	GTTAGTGGCTAAAAAAATCAATCAAGATTACTTGTCGTCATCGTCTTTGTAGTC
AB59	pilA2 qRT F	CCAACAACGCGTTACCTGC
AB60	pilA2 qRT R	TAATCACACCGTTCGCACCA
AB61	pilE qRT F	CCGATGCTCATGCCACATTG
AB62	pilE qRT R	CGTTGTGGCTGTCAGGTTTG
JAC35	hspC2 qRT F	AAATGCCTGGTATGGGCGAA
JAC36	hspC2 qRT R	TCGCTTTATCCACATCCGCA
AL31	lbtA qRT F	CATCGGCCTGTTGGATTAGT
AL32	lbtA qRT R	TCGTGGCATGAAATGGTAGAG
AL37	frgA qRT F	ACTCTCCCGAA TTCCAGGCCAA
AL38	frgA qRT R	TGATTCCTCCACTGCCAAGGGT

* Nucleotides in bold signify the locations of restriction enzymes sites used to facilitate cloning. The corresponding enzymes are listed to the right of the sequences.

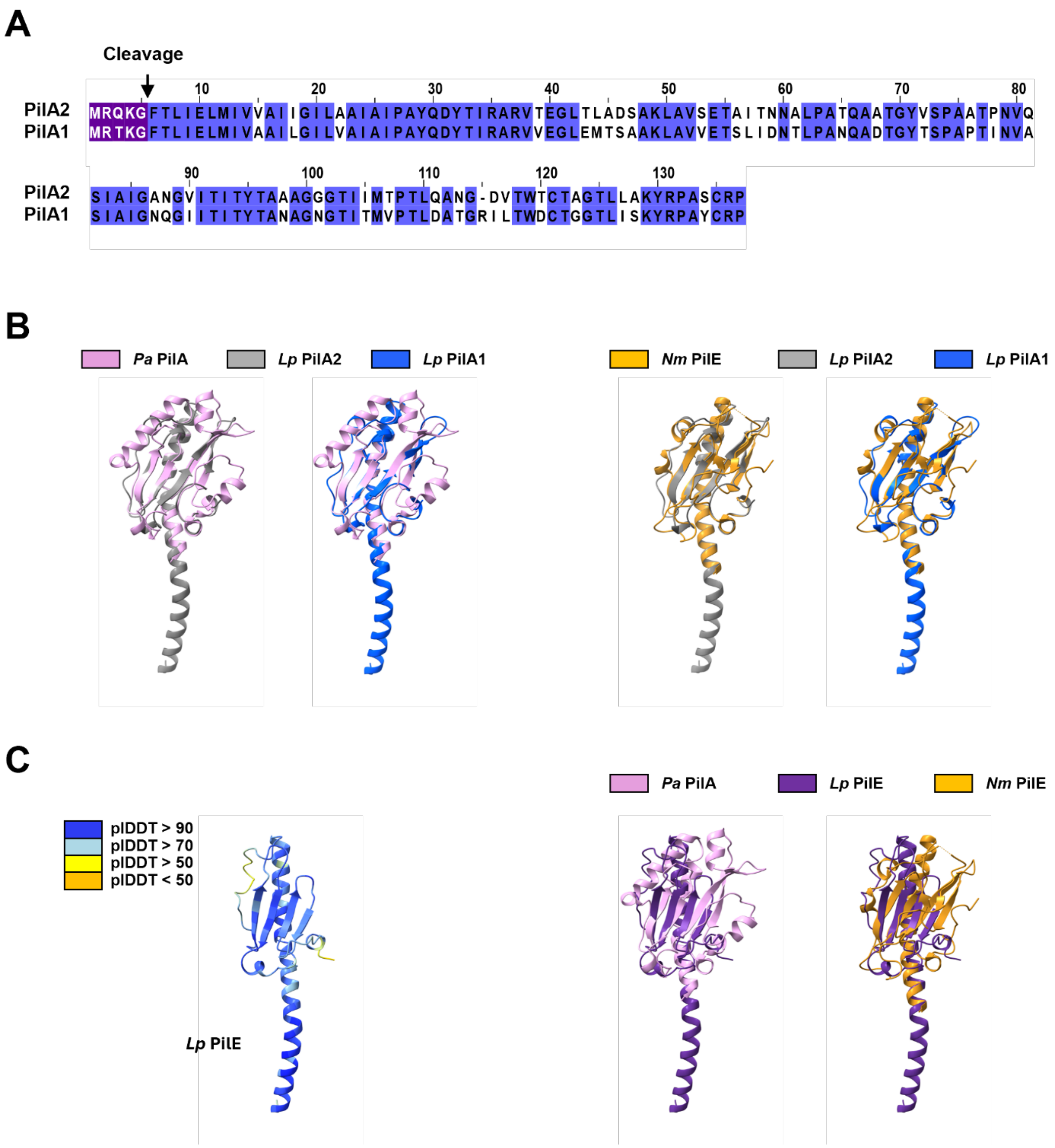

FIG. S1. Sequences of PilA2 and PilA1 and alignments to known T4P major pilins. (A) Amino acid sequence alignment of the PilA2 and PilA1 proteins of *L. pneumophila* strain 130b. Highlighted in purple are the proteins predicted signal sequences followed by their PilD cleavage sites. Regions of sequence identity are shaded in blue. (B) Alignment of the AlphaFold 3-predicted structures of *L. pneumophila* PilA2 (*Lp* PilA2, gray) and PilA1 (*Lp* PilA1, blue) with the known structure of either the major pilin PilA of *P. aeruginosa* (PDB ID: 3JZZ) (*Pa* PilA, pink), giving for PilA2 a RMSD = 2, Z = 10.5, and % identity = 38, and for PilA1 a

RMSD = 2.2, Z = 10, and % identity = 36, or the major pilin PilE of *N. meningitidis* (PDB ID: ID: 4V1J) (*Nm* PilE, orange), giving for PilA2 a RMSD = 2, Z = 12.4, and % identity = 28, and for PilA1 a RMSD = 2.1, Z = 12.2, and % identity = 27. (C) (Left) Predicted 3-D structure of the PilE protein of strain 130b (without its signal sequences) (*Lp* PilE), as discerned by AlphaFold 3. The structure is color-coded in accordance with the levels of confidence (i.e., pLDDT values) determined by the program (upper left). (Right) Alignment of the AlphaFold 3-predicted structure of *L. pneumophila* PilE (*Lp* PilE, purple) with the known structure of either the major pilin PilA of *P. aeruginosa* (*Pa* PilA, pink), giving a RMSD = 2.9, Z = 6.4, and % identity = 9, or PilE of *N. meningitidis* (*Nm* PilE, orange), giving a RMSD = 3.2, Z = 6.8, and % identity = 9. In (B – C), the determined structures of *P. aeruginosa* PilA and *N. meningitidis* PilE lack ~30 N-terminal residues.

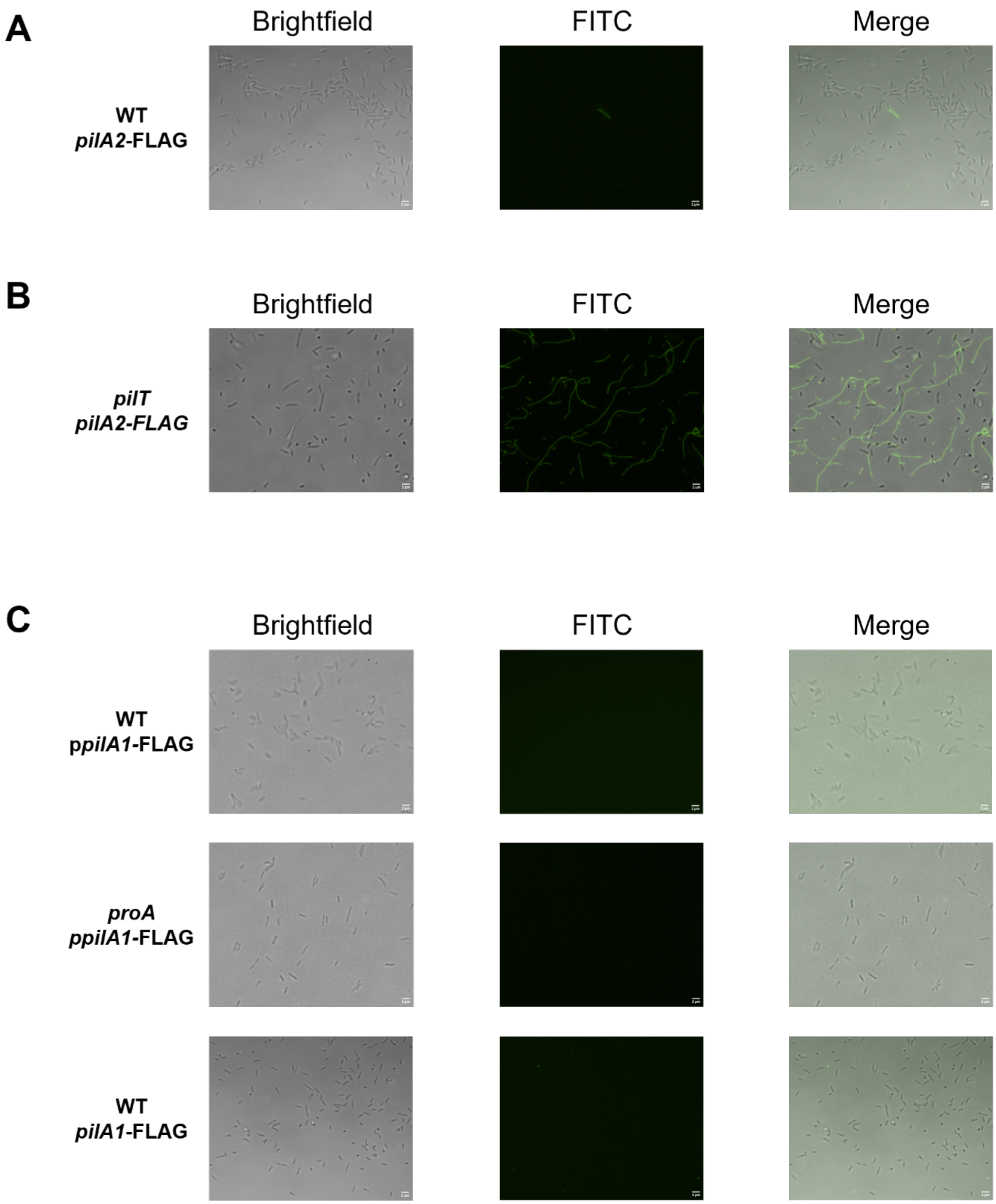

FIG. S2. Expression of chromosomally encoded PilA2 on T4P from WT and *pilT* mutant *L. pneumophila*, and the lack of expression of PilA1 on the *L. pneumophila* surface. (A - B) WT (A) and *pilT* mutant (B) encoding PilA2-FLAG from the native *pilA2* chromosomal locus (WT *pilA2*-FLAG, *pilT pilA2*-FLAG) were grown on BCYE agar for 5 days at 37°C. Bacterial samples were incubated with anti-FLAG antibodies and then examined by IF. Presented are

representative images seen with brightfield or epifluorescence microscopy. In the merged images are bacterial cells with long PilA2-containing pili. (C) WT strain 130b carrying p*pilA1*-FLAG which encodes PilA1-FLAG1 (WT p*pilA1*-FLAG, top row), *proA* mutant AA200 carrying p*pilA1*-FLAG (*proA* p*pilA1*-FLAG, middle row), and WT encoding PilA1-FLAG from its native chromosomal locus (WT *pilA1*-FLAG, bottom row) were grown on BCYE agar at 37°C for 2 days for those strains with plasmid or 5 days for those without plasmid and then examined as described in (A). Although the presented images show ~50 cells of each strain to aid in the visualization of individual cells, no PilA1-containing structures were ever seen despite looking at ~1,000 cells of each strain. For (A – C), the presented images are representative of results from three independent trials.

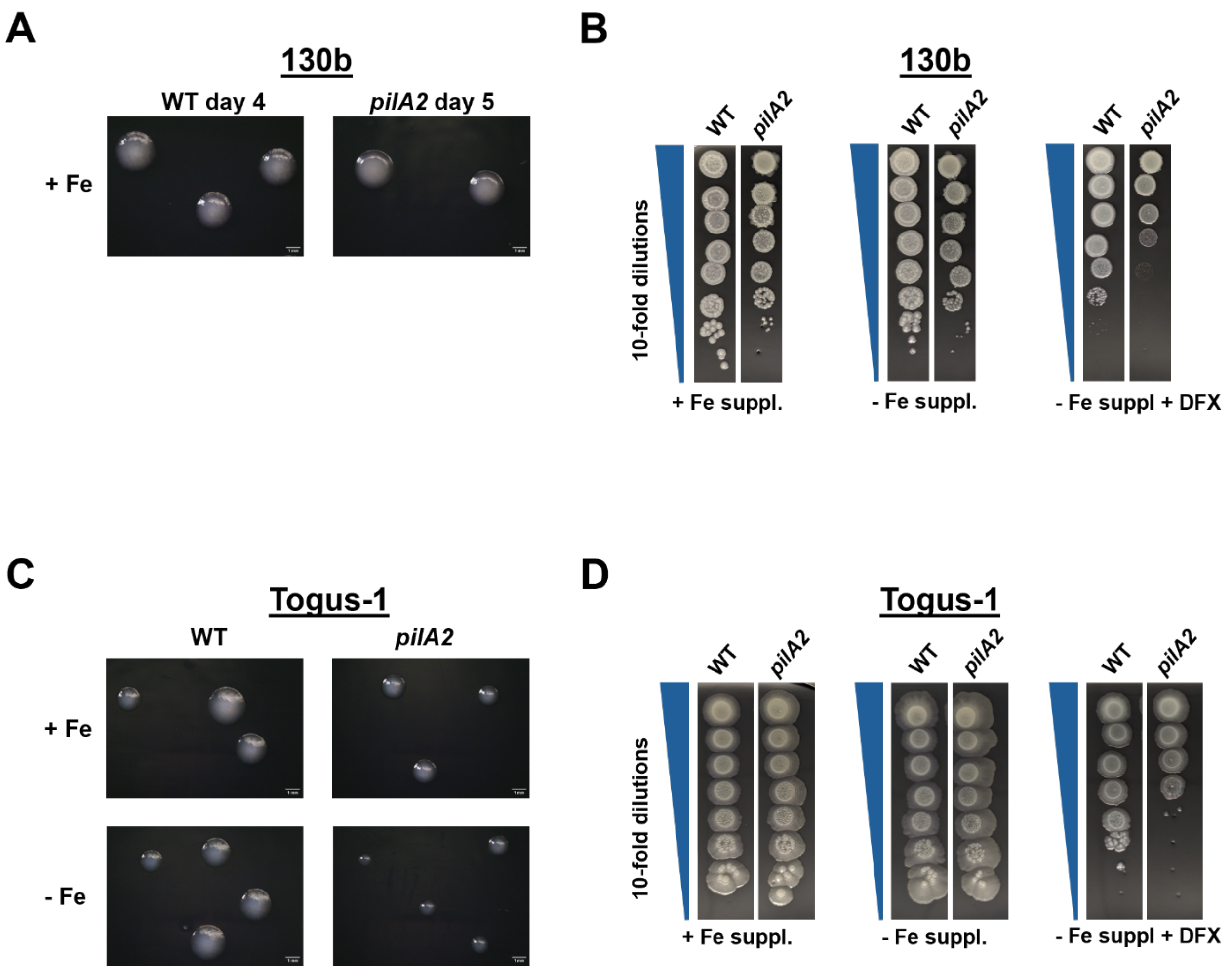

FIG. S3. Further effects of PilA2 on *L. pneumophila* colony size and efficiency of plating on BCYE agar containing varying amounts of iron. (A) WT 130b and *pilA2* mutant NU494 (*pilA2*) were grown for 4 or 5 days on BCYE agar with its standard iron supplement (+ Fe), and images of representative colonies were obtained. (B) WT strain 130b (WT) and *pilA2* mutant NU495 (*pilA2*) were grown for 3 days on BCYE agar and then spotted onto BCYE agar that either contained its standard supplement of ferric pyrophosphate (+ Fe suppl.), lacked that iron supplement (- Fe suppl.), or lacked that supplement but had 10 µM iron chelator DFX added (- Fe suppl. + DFX). Following incubation for 4 days, images were taken of the areas of bacterial growth. (C) WT strain Togus-1 (WT) and its *pilA2* mutant NU502 (*pilA2*) were grown for 3 days on either BCYE agar containing its standard iron supplement (+ Fe) or BCYE agar lacking that supplement (- Fe), and images of representative colonies were obtained. (D) Following growth on BCYE agar for 3 days, Togus WT and Togus *pilA2* were spotted onto either standard BCYE agar (+ Fe suppl.), BCYE agar that lacked its iron supplement (- Fe suppl.), BCYE that lacked that supplement and had 10 µM DFX added (- Fe suppl. + DFX). Following incubation for 7 days, images were taken of the areas of bacterial growth. For (A – D), the data presented are representative of the results from three independent experiments.

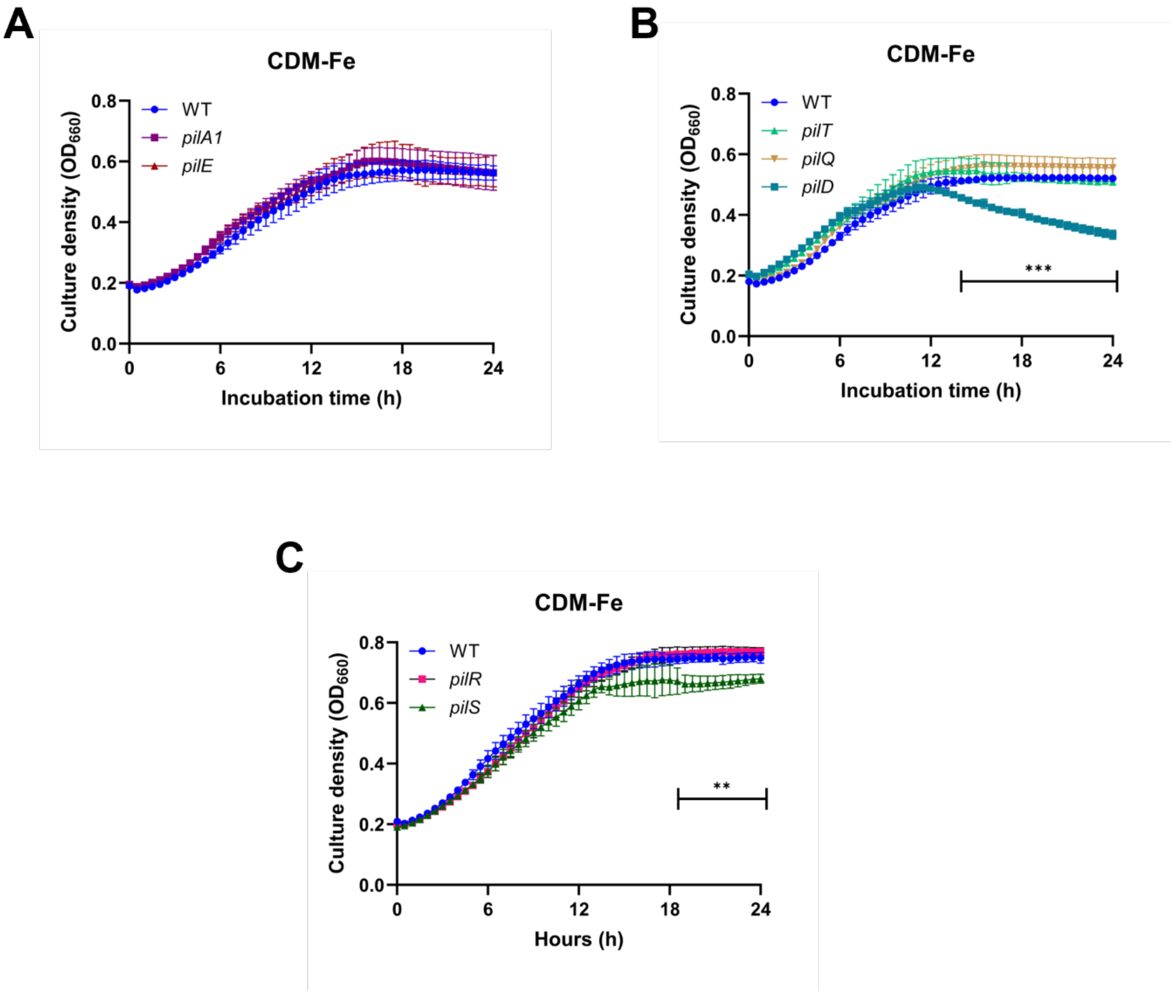

FIG. S4. Growth of T4P-related mutants of *L. pneumophila* in CDM broth lacking added iron. (A - C) WT strain 130b (WT), *pilE* mutant NU496 (*pilE*), and *pilA1* mutant NU497 (*pilA1*) (A), WT, *pilT* mutant NU498 (*pilT*), *pilQ* mutant NU279 (*pilQ*), and *pilD* mutant NU272 (*pilD*) (B), and WT, *pilR* mutant NU501 (*pilR*), and *pilS* mutant NU500 (*pilS*) (C) were grown in CDM-Fe broth (CDM-Fe) in microtiter plates, and optical density readings taken every h for 24 h. For (A – C), the data are presented as the means and standard deviations from three technical replicates. In (B), the *pilD* mutant differs from WT at t = 14 h and beyond, ***, $P < 0.001$. The data in (A – C) are representative of the results from three independent experiments, except for the slight difference (**, $P < 0.01$) between the *pilS* mutant and WT after 18 h in (C), which was not observed in the other two trials done.

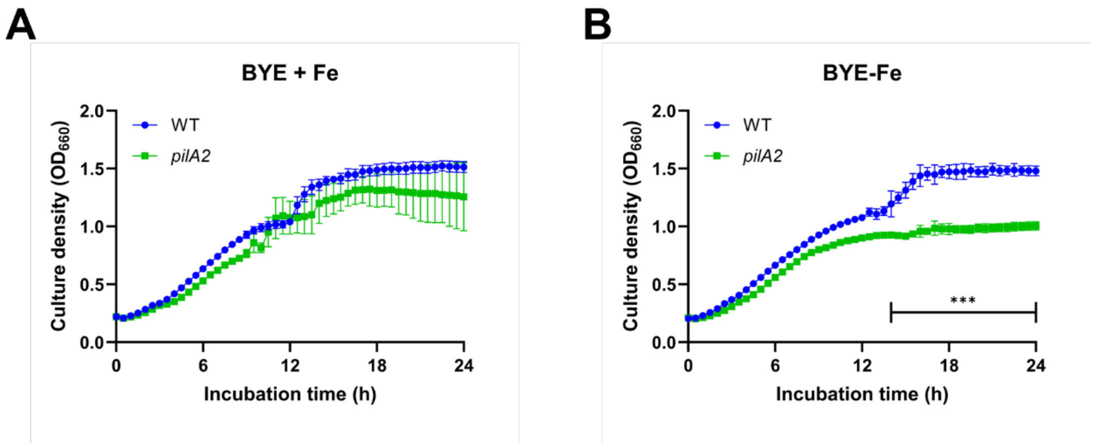

FIG. S5. Growth of *pilA2* mutant *L. pneumophila* at 37°C in BYE broth containing different amounts of added iron. (A – B) WT strain 130b (WT) and *pilA2* mutant NU494 (*pilA2*) were resuspended in either BYE broth containing its standard amount of added iron (BYE + Fe) (A) or BYE broth lacking the iron supplement (BYE – Fe) (B) and added to the wells of a microtiter plate, and then optical density readings obtained every h for 24 h. The data are presented as the means and standard deviations from three technical replicates. In (B), the *pilA2* mutant exhibited impaired growth at t = 14 h and beyond, ***, $P < 0.001$. The data presented in (A – B) are representative of the results from three independent trails.

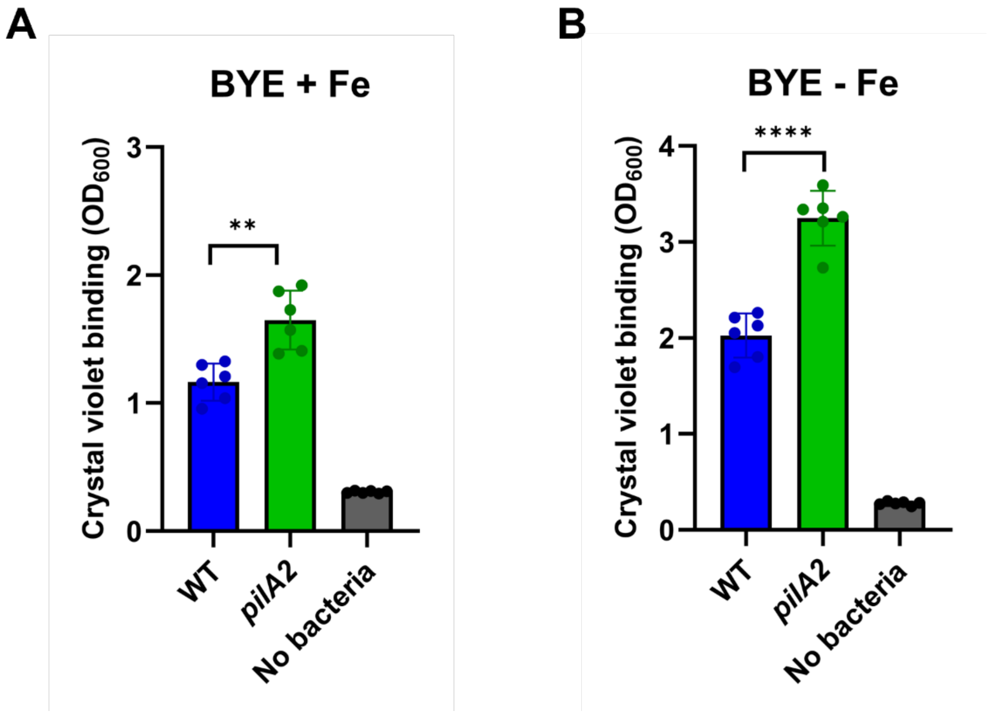

FIG. S6. Effect of PilA2 on *L. pneumophila* biofilm formation at 30°C. (A – B) WT strain 130b (WT) and *pilA2* mutant NU494 (*pilA2*) were resuspended in either BYE broth containing its standard amount of added iron (BYE + Fe) (A) or BYE broth lacking the iron supplement (BYE – Fe) (B) and then the suspensions were added into the wells of a microtiter plate. After 2 d, the amount of biofilm formed was determined by staining with crystal violet as read at 600 nm. Data are presented as means and standard deviations from six technical replicates and are representative of the results from three independent trials. Asterisks indicate the differences in biofilm levels between the *pilA2* mutant and WT, **, $P < 0.01$, ****, $P < 0.0001$.

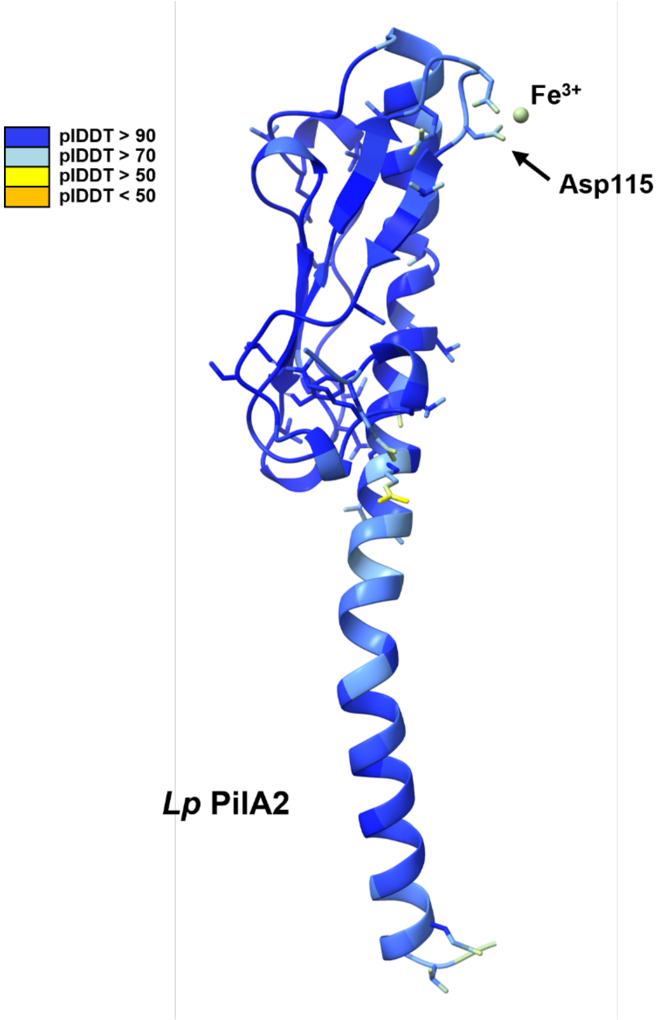

FIG. S7. A possible iron-binding site within *L. pneumophila* PilA2. The AlphaFold 3-predicted structure of PilA2 (without its signal sequence, *Lp* PilA2) is shown in complex with ferric iron at aspartic acid residue-115. The PilA2 structure is color-coded in accordance with its levels of confidence (pLDDT values, upper left), and the image shown here is rotated 90 degrees relative to the image of the PilA2 structure in Fig. 1D. The predicted template modeling (pTM) score for the overall predicted fold for the complexes was 0.85, exceeding the pTM threshold of 0.5. The interface predicted template modelling (ipTM) score that measured the accuracy of the predicted relative positions of subunits in the complex was 0.84, a value higher than the 0.8 threshold for high-quality predictions.
